# Deciphering the Active Compounds and Mechanisms of Qixuehe Capsule on Qi Stagnation and Blood Stasis Syndrome: A Network Pharmacology Study

**DOI:** 10.1155/2020/5053914

**Published:** 2020-02-27

**Authors:** Yu-Xi Huang, Ding-Qiao Xu, Shi-Jun Yue, Yan-Yan Chen, Hui-Juan Tao, Rui-jia Fu, Li-Ming Xing, Taiyi Wang, Yu-ling Ma, Bao-An Wang, Yu-Ping Tang, Jin-Ao Duan

**Affiliations:** ^1^Key Laboratory of Shaanxi Administration of Traditional Chinese Medicine for TCM Compatibility, State Key Laboratory of Research & Development of Characteristic Qin Medicine Resources (Cultivation), Shaanxi Key Laboratory of Chinese Medicine Fundamentals and New Drugs Research, Shaanxi Collaborative Innovation Center of Chinese Medicinal Resources Industrialization, Shaanxi University of Chinese Medicine, Xi'an 712046, China; ^2^Oxford Chinese Medicine Research Centre, University of Oxford, Oxford, UK; ^3^Jiangsu Collaborative Innovation Center of Chinese Medicinal Resources Industrialization, Jiangsu Key Laboratory for High Technology Research of TCM Formulae, National and Local Collaborative Engineering Center of Chinese Medicinal Resources Industrialization and Formulae Innovative Medicine, Nanjing University of Chinese Medicine, Nanjing 210023, China; ^4^Shaanxi Momentum Qixuehe Pharmaceutical Co., Ltd., Xi'an 712000, China

## Abstract

**Background:**

Qixuehe capsule (QXH), a Chinese patent medicine, has been demonstrated to be effective in the treatment of menstrual disorders. In traditional Chinese medicine (TCM) theory, qi stagnation and blood stasis syndrome (QS-BSS) is the main syndrome type of menstrual disorders. However, the pharmacodynamic effect of QXH in treating QS-BSS is not clear, and the main active compounds and underlying mechanisms remain unknown.

**Methods:**

A rat model of QS-BSS was established to evaluate the pharmacodynamic effect of QXH. Thereafter, a network pharmacology approach was performed to decipher the active compounds and underlying mechanisms of QXH.

**Results:**

QXH could significantly reduce the rising whole blood viscosity (WBV) and plasma viscosity (PV) but also normalize prothrombin time (PT), activated partial thromboplastin time (APTT), thrombin time (TT), and fibrinogen (FIB) content in QS-BSS rats. Based on partial least-squares-discriminant analysis (PLS-DA), the low-dose QXH-intervened (QXH-L) and the high-dose QXH-intervened (QXH-H) groups seemed the most effective by calculating the relative distance to normality. Through network pharmacology, QXH may improve hemorheological abnormality mainly via 185 compounds-51 targets-28 pathways, whereas 184 compounds-68 targets-28 pathways were associated with QXH in improving coagulopathy. Subsequently, 25 active compounds of QXH were verified by UPLC-Q/TOF-MS. Furthermore, 174 active compounds of QXH were shared in improving hemorheological abnormality and coagulopathy in QS-BSS, each of which can act on multiple targets to be mainly involved in complement and coagulation cascades, leukocyte transendothelial migration, PPAR signaling pathway, VEGF signaling pathway, and arachidonic acid metabolism. The attribution of active compounds indicated that Angelicae Sinensis Radix (DG), Paeoniae Radix Rubra (CS), Carthami Flos (HH), Persicae Semen (TR), and Corydalis Rhizoma (YHS) were the vital herbs of QXH in treating QS-BSS.

**Conclusion:**

QXH can improve the hemorheology abnormality and coagulopathy of QS-BSS, which may result from the synergy of multiple compounds, targets, and pathways.

## 1. Introduction

Qixuehe capsule (QXH) is a Chinese patent medicine and used clinically for the treatment of menstrual disorders for a dozen years. Menstrual disorders are known to be a common gynecological disease, which is characterized by abnormal menstrual cycle, dysmenorrhea, amenorrhea, uterine bleeding, postpartum abdominal pain, lochia, breast swelling, infertility, and chloasma, seriously affecting female's health [1, 2]. Although the symptoms of menstrual disorders are complex and diverse, qi stagnation and blood stasis syndrome (QS-BSS) is the main syndrome type of menstrual disorders in traditional Chinese medicine (TCM) theory. QS-BSS is defined as due to the poor stagnation of the operation of the qi, the blood is running impediment and the pathological state of blood stasis occurs, which is mainly related to menstrual disorders, hyperlipidemia, liver injury, ischemic brain injury, and hypertension [3–5]. The formation of QS-BSS is accompanied by the following pathological changes: (1) abnormal hemorheology; (2) increased blood coagulability or reduced fibrinolytic activity; (3) microcirculation disturbance; (4) increased platelet aggregation or decreased release function; (5) hemodynamic disturbance; (6) manifestations of static blood in pathological section; (7) vessel obstruction [6]. Numerous studies have shown that the pathological changes of hemorheological abnormality and coagulopathy are more important during the formation of QS-BSS [7, 8]. TCM used to treat syndrome by compound formulas, which are composed of several or even tens of herbal medicines in different quantities, following the composition theory “Monarch, Minister, Assistant and Guide” [9]. QXH is based on the addition and subtraction of Xuefu Zhuyu decoction (a classic blood-activating formula) and Chaihu Shugan powder (a classic qi-regulating formula), which consists of Angelicae Sinensis Radix (DG), Paeoniae Radix Rubra (CS), Chuanxiong Rhizoma (CX), Persicae Semen (TR), Bupleuri Radix (CH), Carthami Flos (HH), Salviae Miltiorrhizae Radix et Rhizoma (DS), etc., so as to regulate liver-qi stagnation, promote blood circulation, and remove blood stasis in the treatment of QS-BSS [10, 11]. However, the potential active compounds and underlying mechanisms of QXH in treating QS-BSS remain unclear.

In recent years, network pharmacology has been proposed as a promising approach for understanding compound formulas [12, 13]. Studies have shown that Xuefu Zhuyu decoction and compound Danshen formula have been analyzed by network pharmacology, and the main active compounds in the formulas and the treatment mechanisms have been elucidated [14, 15]. Therefore, network pharmacology is promising to decipher the active compounds and possible mechanisms of QXH.

**Figure 1:**
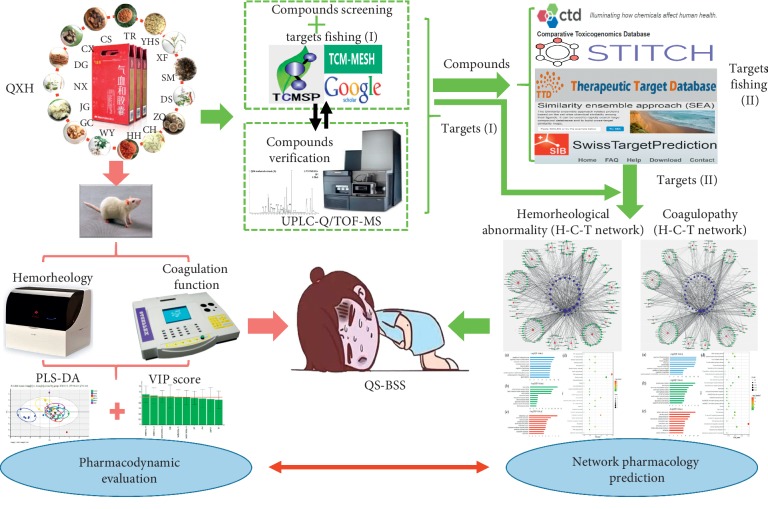
The workflow of QXH in treating QS-BSS combined with pharmacodynamic evaluation and network pharmacology prediction.

In this study, we evaluated the pharmacodynamic effect of QXH in the treatment of QS-BSS based on animal experiments from the two aspects: hemorheology and coagulation function. Hemorheological abnormality is reflected in changes in blood fluidity, deformability, and viscosity, which can be measured by whole blood viscosity (WBV) and plasma viscosity (PV). Meanwhile, coagulopathy is reflected in the abnormality of coagulation factor function; thrombin time (TT), prothrombin time (PT), activated partial thromboplastin time (APTT), and fibrinogen content (FIB) are the important monitoring index of coagulopathy in clinical aspects [16]. Subsequently, a network pharmacology approach was performed to decipher the active compounds of QXH and its regulation mechanisms of QS-BSS. By constructing two herb-compound-pathological process relationship diagrams, the possible mechanisms of QXH in the treatment of QS-BSS were systematically elucidated. A workflow of the entire study procedure is depicted in Figure 1.

## 2. Materials and Methods

### 2.1. Drugs and Reagents

QXH (Lot. 161030) was supplied by Shaanxi Momentum Pharmaceutical Co., Ltd., Shaanxi, China; compound danshen dripping pills (CDDP, Lot. 180602) were purchased from Tasly Pharmaceutical Co., Ltd., Tianjin, China; and adrenaline hydrochloride injection (Adr, Lot. 20190102) was obtained from Grand Pharmaceutical Co., Ltd., Wuhan, China. Carboxymethylcellulose sodium (Lot. 20180803) was purchased from Tianli Chemical Reagent Co., Ltd., Tianjin, China; and disposable human venous blood sample collection containers (3.2% sodium citrate anticoagulation, blood/citrate: 9 : 1, v/v, Lot. 20190407, and heparin sodium anticoagulation, Lot. 20190705) were obtained from Yuli Medical Instrument Co., Ltd., Jiangsu, China. PT (Lot. 20190214), APTT (Lot. 20190214), TT (Lot. 20190114), and FIB (Lot. 20192012) assay kits were obtained from Steellex Biotechnology Co., Ltd., Taizhou, China. MS-grade acetonitrile and methanol were purchased from Merck, Darmstadt, Germany. MS-grade formic acid was obtained from Sigma-Aldrich, Darmstadt, Germany. Water used in the experiment was purified by using a Milli-Q water purification system (Billerica, MI, USA). Reference substances were purchased from Yuanye Bio-Technology Co., Ltd., Shanghai, China. The purity of each compound was determined to be higher than 98% by HPLC.

### 2.2. Animal Handling

Female Sprague-Dawley rats (200 ± 20 g) were provided by Dossy Experimental Animals Co., Ltd., Chengdu, China. All procedures were conducted in accordance with the Regulations of Experimental Animal Administration issued by the State Committee of Science and Technology of the People's Republic of China and approved by the Animal Ethics Committee of Shaanxi University of Chinese Medicine.

After acclimatization for one week, the rats were randomly divided into 6 groups with 8 rats in each group: control, model, low-dose QXH-intervened (QXH-L), middle-dose QXH-intervened (QXH-M), high--dose QXH-intervened (QXH-H), and CDDP-intervened (CDDP, the positive control) groups. The models QXH-L (clinical equivalent does, 0.432 g·kg^−1^), QXH-M (3 times clinical equivalent does, 1.296 g·kg^−1^), QXH-H (6 times clinical equivalent does, 2.592 g·kg^−1^), and CDDP (clinical equivalent does, 0.1 g·kg^−1^) groups were hypodermically injected with Adr 0.9 mg·kg^−1^·d^−1^, and 4 h later, the rats were received chronic unpredictable stimulations (A: 2 h ultrasound stimulation at days 1, 7, and 12; B: day and night upside down at days 2, 9, and 14; C: 2 h physical restraint at days 3, 6, and 11; D: 0–2°C ice swim for 4 min at days 4, 8, and 10; E: 10 min tail suspension at days 5 and 10) to establish the QS-BSS rat model within 14 days. The drug dose conversion formula was as follows: human dose of crude herbs in clinic × 0.018/200 × 1000 × the multiple of clinical equivalent dose [17]. QXH and CDDP samples were dissolved in 0.5% carboxymethylcellulose sodium solution for intragastric administration. Based on the results of preexperiment, the rats in QXH and CDDP treatment groups were intragastrically given the corresponding dose of QXH and CDDP daily from the 7^th^ day, whereas the rats in the control and model groups were intragastrically given the same volume of 0.5% carboxymethylcellulose sodium solution [18].

### 2.3. Sample Collection and Indicator Measurements

On the 14^th^ day, after subcutaneous injection of Adr, the rats were managed to be fasting but had free access to clean water. The next morning at 8 : 00, rats were anesthetized with 10% chloral hydrate (350 mg/kg i.p.). Thereafter, 4 mL blood samples of rats were collected by the abdominal aorta intubation approach into a 5 mL disposable human venous blood sample collection container (heparin sodium anticoagulation). Then, 800 *μ*L of whole blood was used to measure WBV at the shear rate of 1 s^−1^, 5 s^−1^, 50 s^−1^, 100 s^−1^, and 200 s^−1^, and 800 *μ*L plasma was separated from the rest of whole blood by centrifugation (3H16RI refrigerated centrifuge, Herexi Instrument & Equipment Co., Ltd., Hunan, China) at 3000 rpm for 10 min to detect PV by using a SA-5600 automatic hemorheology analyzer (Succeeder Technology Co., Ltd., Beijing, China). Meanwhile, blood samples were collected by the abdominal aorta intubation approach into a 2 mL disposable human venous blood sample collection container (3.2% sodium citrate anticoagulation, blood/citrate: 9 : 1, v/v), and centrifuged at 3000 rpm for 10 min immediately. 500 *μ*L plasma was used to test coagulation function (PT/s, APTT/s, TT/s and FIB/g/L) by using a SC40 semiautomatic coagulation analyzer (Steellex Biotechnology Co., Ltd., Taizhou, China).

### 2.4. QXH Database Construction

The chemical compounds of 15 herbs of QXH were data-mined from relevant databases including Traditional Chinese Medicine Systems Pharmacology Database and Analysis Platform (TCMSP, http://lsp.nwu.edu.cn/index.php) [19] and TCM-MESH (http://mesh.tcm.microbioinformatics.org), and all candidate compounds were also related to the literature to determine the attribution relationship with herbs. Latin names, the number of active compounds of each herb in QXH involving in mediating hemorheological abnormality and coagulopathy, and the corresponding abbreviations of each herb are displayed in Table 1.

### 2.5. Active Compound Screening

Generally, a TCM formula covers various compounds, and bioactive compounds can contribute to its therapeutic effects. In order to obtain the potential active compounds from these herbs of QXH, we applied a method incorporating oral bioavailability (OB), Caco-cell permeability (Caco-2), and drug likeness (DL) evaluation in this work [20, 21].

OB is an important pharmacokinetic parameter which describes the ratio of the amount absorbed into systemic circulation when dosed orally compared to the amount in blood upon intravenous dosing. In the present study, the OB values were evaluated using the robust *in silico* tool OBioavail1.1 [22]. The compounds with OB ≥ 30% were assessed as active compounds for further analysis. Caco-2 is another vital parameter frequently used as a model for studying the passive diffusion of drugs across the intestinal epithelial barrier. Thus, the i*n silico* Caco-2 permeability model which was constructed by 100 drug molecules with satisfactory statistical results (*R*^2^ > 0.8) was employed to select compounds that are more likely to possess good permeability [23]. Given those compounds with Caco-2 value less than −0.4 not permeable, the threshold of Caco-2 permeability is set at −0.4.

DL is a qualitative property of chemicals that describes how the pharmacokinetic and pharmaceutical properties of compounds, such as solubility and potency, correspond in the majority of known drugs. In order to identify drug-like compounds, we applied a database-dependent model using the Tanimoto coefficient [24] to calculate the DL (see equation (1)) of each compound in QXH:

(1) $$\begin{array}{c} T\left(A,B\right)=\frac{A\times B}{{\left|A\right|}^{2}+{\left|B\right|}^{2}-A\times B}. \end{array}$$

In equation (1), *A* represents the molecular descriptors of herbal compounds and *B* displays the average molecular properties of all compounds in Drugbank. Those compounds with DL ≥ 0.18 were preserved. The compounds were adopted as the candidate compounds for further analysis when they met all these three criteria.

Besides, due to the profound pharmacological effects, biological activity, and high contents, those compounds with low OB, Caco-2, or DL values were also selected for further analysis ([Supplementary-material supplementary-material-1]). Last but not least, all active compounds were imported into PubChem (https://pubchem.ncbi.nlm.nih.gov) [25] and ChemSpider (http://www.chemspider.com) [26] to standardize their English names.

### 2.6. Sample Preparation and UPLC-Q/TOF-MS Verification

Reference standards were accurately weighed and directly prepared in methanol as individual stock solutions; subsequently, the stock solutions of twelve standards were continuously diluted with methanol to obtain a suitable concentration for further analysis, respectively. QXH samples were divided into two. 1.0000 g QXH powder was accurately weighed for each and extracted with methanol (25 mL) and water (25 mL) in ultrasonic water bath (40 kHz, 500 W) for 30 min at room temperature, respectively. Subsequently, the QXH methanol and water extracts were centrifuged at 14,000 rpm for 10 min, and the supernatant was stored at 4°C until analysis.

The UPLC-Q/TOF-MS system consisted of an Acquity™ ultra-performance liquid chromatography (UPLC) system (Waters Corporation, Milford, MA, United States) and a Synapt G2 mass spectrometer (MS) (Waters MS-Technologies, Manchester, United Kingdom) equipped with an electrospray ion (ESI) source. The system and data were controlled by MassLynx (V4.1) software. Gradient elution was performed on an ACQUITY UPLC BEH C18 column (2.1 mm × 100 mm, 1.7 mm; Waters) at 35°C. The optimal mobile phase was composed of 0.1% formic acid aqueous solution (A) and acetonitrile (B): 0–2 minutes, maintained at 2% B; 2–4 minutes, 2–20% B; 4–5 minutes, 20–45% B; 5–8 minutes, 45–48%; 8–10 minutes, 48–75% B; 10–13 minutes, 75–90% B; 13–19 minutes, maintained at 90% B; 19–22 minutes, 90–95% B; 22–23 minutes, 95–20% B; 23–24 minutes, 20–2% B; 24–25 minutes, maintained at 2% B. A 2 *μ*L aliquot of sample solution was injected for analysis, and the flow rate was 0.4 mL/min.

The full-scan LC-MS data were acquired in both positive and negative ion modes from 100 to 1300 Da with a 0.3 s scan time. The optimal Q/TOF-MS conditions were as follows: capillary voltage 3 kV, sampling cone voltage 40.0 V, and extraction cone voltage 4.0 V for positive ion mode; capillary voltage 2.5 kV, sampling cone voltage 40.0 V, and extraction cone voltage 4.0 V for negative ion mode. The source temperature and desolvation gas temperature were set to 100 and 350°C, respectively, and the cone gas flow and desolvation gas flow were set to 50 L/h and 600 L/h, respectively. The collision energy was set to 4 eV for positive ion mode and 2 eV for negative ion mode. Sodium formate solution was used to calibrate the mass spectrometer prior to the experiment. Leucine-enkephalin was used as an external reference (Lock-Spray™) to correct the mass during data acquisition via a LockSpray interface, generating reference ions at *m*/*z* 556.2771 Da ([*M* + *H*]^+^) in the positive ion mode and *m*/*z* 554.2615 Da ([*M* − H]^−^) in the negative mode.

### 2.7. Target Prediction

In order to identify the corresponding targets of active compounds in QXH, several methods combined with chemometric strategy, database dependent, information integration, and literature-mining were implemented [27]. Firstly, all active compounds were imported into TCMSP and TCM-MESH again to obtain relevant targets. Subsequently, all active compounds were imported into STITCH (http://stitch.embl.de/) [28], Therapeutic Targets Database (TTD, http://bidd.nus.edu.sg/group/ttd/) [29], Comparative Toxicogenomics Database (CTD, http://ctdbase.org/) [30], and Google Scholar to obtain H-C-T. In view of the preliminary mining results, the active compounds with less targets were introduced into the Similarity Ensemble Approach (SEA, http://sea.bkslab.org/) [31] and Swiss Target Prediction (http://www.swisstargetprediction.ch) [32] to predict their possible targets. Given the prediction results with Max TC < 0.57 and Possibility < 0 not considered as probably having affinity to the appropriate target, the targets with Max TC > 0.57 in SEA and Possibility > 0 in Swiss Target Prediction were kept in the result, respectively. Thereafter, for the purpose of dissecting the mechanisms of the candidate targets in regulating the pathological processes of QS-BSS, the target information was submitted to TTD, CTD, Online Mendelian Inheritance in Man Database (OMIM, http://www.omim.org/) [33], and Uniprot (https://www.uniprot.org) [34] to determine the targets which were related to hemorheological abnormality and coagulopathy, respectively. Finally, all the targets from the previous mining were imported into Uniprot to obtain their corresponding gene symbol, and only the targets of *Homo sapiens* were kept for further analysis.

### 2.8. Gene Ontology Enrichment and KEGG Pathway Analysis

The Database for Annotation, Visualization and Integrated Discovery (DAVID, http://david.abcc.ncifcrf.gov) web server was employed to perform gene ontology enrichment and KEGG pathway analysis for the targets related to hemorheological abnormality and coagulopathy, respectively [35]. Meanwhile, gene ontology enrichment and KEGG pathway analysis were also performed for the overlapped targets involving in the pathological processes of hemorheological abnormality and coagulopathy.

### 2.9. Network Construction

The herb-active compound-target (H_H_-C_H_-T_H_) and herb-active compound-target (H_C_-C_C_-T_C_) networks of QXH were constructed in the hemorheological abnormality module and coagulopathy module, respectively. All networks were generated and analyzed by Cytoscape 3.6.0 (http://www.cytoscape.org/), an open-source software package project for visualizing, integrating, modeling, and analyzing the interaction networks [36].

### 2.10. Data Processing and Statistical Analysis

All LC-MS and MS/MS data were processed with MassLynx™ (V4.1, Waters, Manchester, UK) software. Molecular formula speculations of the compounds were determined with Elemental Composition software. Then, the acquired raw data matrices on hemorheology and coagulation function were imported to SIMCA-P (version 14.1, Umetrics, Sweden) for principal component analysis (PCA) and partial least-squares-discriminant analysis (PLS-DA). PCA is an unsupervised multivariate statistical approach used for variable reduction and separation into classes. To maximize class discrimination, the data were further analyzed utilizing PLS-DA [16, 37]. The parameters of PCA (*R*^2^*X* and *Q*^2^) and PLS-DA (*R*^2^*X*, *R*^2^*Y* and *Q*^2^) were used to evaluate the model, and *Q*^2^ is an appraisal criterion of the predictive ability of a model. *R*^2^*X* represents the capability wherein the primary components of variables are used to build the model and sample, whereas *R*^2^*Y* represents the conformity level of the sample with the model. Then, the PLS-DA model was validated by 200-repeated permutation tests and CV-ANOVA (analysis of variance testing of cross-validated predictive residuals) tests. A model with the *p* value (obtained by CV-ANOVA) below 0.5 or with *R*^2^ and *Q*^2^ values (obtained by permutation tests) lower than the values of the model itself was considered to be valid. The value of variable importance in the projection (VIP) from the PLS-DA model represents the contribution of variables to the classification of the model which was analyzed. The differential parameters between the control and the model groups were selected by VIP >1. All data were expressed as mean ± standard deviation (SD) and analyzed by one-way ANOVA in SPSS 16.0 (SPSS Inc., Chicago, IL, USA). The criterion for statistical significance was *p* < 0.05.

## 3. Results

### 3.1. Effects of QXH on Hemorheology in Rats with QS-BSS

QS-BSS mainly occurs with increased blood viscosity and poor blood fluidity. WBV and PV are the vital hemorheological parameters and commonly used to evaluate the clinical therapeutic efficacy of drugs. In Table 2, the levels of WBV in low and high shear rates (1 s^−1^, 5 s^−1^, 50 s^−1^, 100 s^−1^, and 200 s^−1^) and PV were significantly elevated in the model group in comparison with the control group (*p* < 0.01), indicating a successful modeling. Compared with the model group, the WBV at the shear rate of 1 s^−1^, 5 s^−1^, 50 s^−1^, 100 s^−1^, and 200 s^−1^ in QXH and CDDP groups significantly decreased near to normality (*p* < 0.05 or *p* < 0.01). Meanwhile, QXH and CDDP groups could significantly decrease the rising PV in QS-BSS rats (*p* < 0.01), suggesting that both QXH and CDDP treatment could significantly improve the hemorheological abnormality in rats with QS-BSS, and QXH is no less effective than CDDP in improving hemorheology in QS-BSS rats.

### 3.2. Effects of QXH on Coagulation Function in Rats with QS-BSS

APTT, PT, TT, and FIB, as coagulation function test items in clinics, are crucial quantitative indexes of pathological changes in the coagulation system of the body [36]. As is illustrated in Table 3, compared with the control group, TT, PT, and APTT levels were significantly shortened (*p* < 0.05 or *p* < 0.01) and FIB content was significantly elevated (*p* < 0.01) in the model group. Compared with the model group, the level of TT, PT, APTT and FIB in QXH groups had different degrees of callback. Specifically, QXH-L treatment could significantly rectify the perturbed TT and PT after QS-BSS modeling (*p* < 0.05); the QXH-M group significantly improved the abnormality of TT and APTT (*p* < 0.05 or *p* < 0.01); the QXH-H group showed a significantly effect in improving TT (*p* < 0.01). CDDP significantly brought the disrupted TT, APTT, and FIB after QS-BSS modeling to near normality (*p* < 0.05 or *p* < 0.01). All above results fully prove that QXH is comparable with CDDP on improving coagulation function in QS-BSS rats.

### 3.3. Evaluation of the Effects of QXH on Improving Hemorheological Abnormality and Coagulopathy Based on Multivariate Statistical Analysis

**Figure 2:**
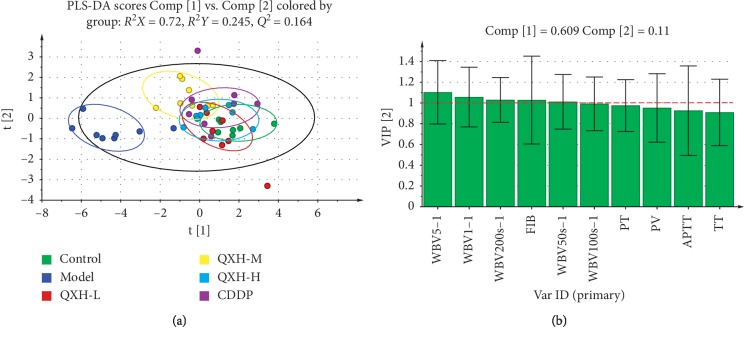
(a) PLS-DA score plot of hemorheology and coagulation function indexes among all groups. (b) VIP score plot of hemorheology and coagulation function indexes between control and model groups. WBV200 s^−1^: whole blood viscosity in 200 s shear rate; WBV100 s^−1^: whole blood viscosity in 100 s shear rate; WBV50 s^−1^: whole blood viscosity in 50 s shear rate; WBV5 s^−1^: whole blood viscosity in 5 s shear rate; WBV1 s^−1^: whole blood viscosity in 1 s shear rate; PV: plasma viscosity; APTT: activated partial thromboplastin time; PT: prothrombin time; TT: thrombin time; FIB: fibrinogen content.

In this study, PCA was used firstly to determine the general interrelation between control, model, QXH-L, QXH-M, QXH-H, and CDDP groups. As is shown in [Supplementary-material supplementary-material-1], the model group and other groups were separated clearly, indicating that the rats of the model group have significantly altered on hemorheology and coagulation function. The parameters of the PCA model: *R*^2^*X* = 0.982; *Q*^2^ = 0.582 ([Supplementary-material supplementary-material-1]) suggesting good ability of prediction and reliability of the model, and the variances of Comp [1] and Comp [2] account for 0.61 and 0.125, respectively. To provide better visualization for discriminating groups of samples from PCA and for class-separating information of the variables, a supervised approach (PLS-DA) was performed. Obviously, in a PLS-DA score plot (Figure 2), the center of the control group is about (1.69, −0.4), the center of the model group is about (−4.36, −0.61), the center of the QXH-L group is about (0.67, −0.46), the center of the QXH-M group is about (−0.65, 1.07), the center of the QXH-H group is about (0.61, 0.08), and the center of the CDDP group is about (1.03, 0.38). By the analysis of central distance among groups, it was suggested that the QXH-L and QXH-H groups have a better pharmacodynamic effect on QS-BSS. Meanwhile, the CDDP group also shows powerful regulation on rats with QS-BSS to the normal level. The parameters of the PLS-DA model: *R*^2^*X* = 0.72, *R*^2^*Y* = 0.245, and *Q*^2^ = 0.164 (Figure 2(a)), suggesting the goodness of fit to data. And the variances of Comp [1] and Comp [2] in Figure 2 account for 0.609 and 0.11, respectively. Permutation tests (*n* = 200) and CV-ANOVA tests were used to validate the PLS-DA model among all groups ([Supplementary-material supplementary-material-1]) and the PLS-DA model with *R*^2^ = (0.0, −0.0123), *Q*^2^ = (0.0, −0.191), and *p*=0.000490249, indicating that the PLS-DA model is valid.

Subsequently, the second round of the PLS-DA method was used to analyze the VIP value of each index between control and model groups to obtain the most contribution indexes to the two groups' differences. The parameters of PLS-DA between control and model groups: *R*^2^*X* = 0.884, *R*^2^*Y* = 0.918, and *Q*^2^ = 0.87. These results indicated the excellent reliability and predictive capability of the model ([Supplementary-material supplementary-material-1]). The results of 200 permutations tests and CV-ANOVA tests on the PLS-DA model with *R*^2^ = (0.0, 0.27), *Q*^2^ = (0.0, −0.196), and *p*=0.000174217 (Figures [Supplementary-material supplementary-material-1]) indicate the PLS-DA model is valid. As is shown in Figure 2(b), the value of VIP from the PLS-DA model represents the contribution of variables of the indexes to the classification between control and model groups. The larger the VIP value, the greater the contribution of those indexes to the difference between control and model groups. The VIP value of WBV5 s^−1^, WBV1 s^−1^, WBV200 s^−1^, FIB, and WBV50 s^−1^ are all >1, indicating WBV and FIB are vital to distinguish the difference between normal and QS-BSS levels and can be used as the essential indexes to evaluate the pharmacodynamic effect of QXH in the treatment of QS-BSS. The VIP value of TT and APTT are about 0.9, suggesting the poor contribution to the difference between control and model groups.

### 3.4. Active Compounds of QXH

To better understand the molecular mechanisms behind the therapeutic effects of QXH, a total of 195 active compounds were selected for further analysis, 25 of which were verified in QXH by UPLC-Q/TOF-MS, such as tetrahydropalmatine (S1), berberine (S2), butylated hydroxytoluene (S3), tangeretin (S4), *β*-sitosterol (S5), Z-ligustilide (S6), cryptotanshinone (S7), tanshinone I (S8), levistilide A (S9), tanshinone IIA (S10), stigmasterol (S11), oxypaeoniflorin (S12), amygdalin (13), caffeic acid (S14), albiflorin (S15), paeoniflorin (S16), liquiritin (S17), ferulic acid (S18), isoferulic acid (S19), rosmarinic acid (S20), liquiritigenin (S21), benzoylpaeoniflorin (S22), glycyrrhizic acid (S23), isoliquiritigenin (S24), and glycyrrhetic acid (S25) ([Supplementary-material supplementary-material-1], Figures [Supplementary-material supplementary-material-1] and [Supplementary-material supplementary-material-1]). Especially, butylated hydroxytoluene, berberine, tangeretin, quercetin, luteolin, columbamine, ellagic acid, and tanshinone IIB are the major agents of QXH, which play anti-inflammation, antiatherosclerotic, antiplatelet aggregation, and antithrombotic effects, respectively [38, 39]. It was worth noting that although several compounds have relatively low pharmacokinetic values, they were either the richest compounds of the herbs or biologically active, like paeonolide, danshensu, and rosmarinic acid; thus, those compounds were also considered as candidate compounds for further analysis. In other words, there were 19 compounds in Angelicae Sinensis Radix (DG), 17 compounds in Paeoniae Radix Rubra (CS), 15 compounds in Chuanxiong Rhizoma (CX), 12 compounds in Persicae Semen (TR), 15 compounds in Carthami Flos (HH), 11 compounds in Bupleuri Radix (CH), 11 compounds in Cyperi Rhizoma (XF), 29 compounds in Salviae Miltiorrhizae Radix et Rhizoma (DS), 33 compounds in Corydalis Rhizoma (YHS), 6 compounds in Platycodonis Radix (JG), 14 compounds in Aurantii Fructus (ZQ), 7 compounds in Linderae Radix (WY), 13 compounds in Achyranthis Bidentatae Radix (NX), f 19 compounds in Cimicifugae Rhizoma (SM), and 26 compounds in Glycyrrhizae Radix et Rhizoma (GC). *Z*-ligustilide, senkyunolide A, senkyunolide I, 3-butylidenephthalide, coniferyl ferulate, vanillin, erulic acid, and sedanolide were shared in DG and CX. Quercetin was shared in HH, NX, WY, YHS, CH, XF, and ZQ. Rosmarinic acid was shared in HH and DS. Kaempferol was shared in HH, NX, CH, XF, and ZQ.

### 3.5. Target Identification

Presently, for QXH, altogether 102 proteins were identified as its targets, and 51 of 102 targets were involved in hemorheological abnormality module, 68 of 102 targets were involved in coagulopathy module, and 17 targets were shared in improving hemorheological abnormality and coagulopathy. In the hemorheological abnormality module, PTGS1, PTGS2, AR, ESR1, ESR2, CHRM5, PIK3CG, MAPK14, CYP3A4, and HMGCR were included. PTGS1 and PTGS2, two isoforms of cyclooxygenase, are the anti-inflammation targets of most NSAIDs [40], PTGS1 is constitutively expressed in various cells and tissues, fulfilling housekeeping functions, such as vascular homeostasis and platelet aggregation [41]. PTGS2 is an immediate-early response gene induced in response to mitogens, tumor promoters, cytokines, growth factors, and inflammatory stimuli [42]. ESR1 is mainly expressed in macrophages, endothelial cells, and vascular smooth muscle cells, and it plays an important role in vascular wall physiology and function. Single-nucleotide polymorphisms in the ESR1 gene have been associated with vascular diseases and conditions, such as arterial hypertension, CVD, cerebrovascular disease, and serum lipid level alterations [43]. In the coagulopathy module, besides PTGS1, PTGS2, and PIK3CG, F2, F10, PRKACA, NOS3, PTPN2, NQO1, and HMOX1 were also included. F2 and F10 cleave bonds after Arg and Lys, convert fibrinogen to fibrin and activate factors V, VII, VIII, and XIII, in combination with thrombomodulin and protein C, and has function in blood homeostasis, inflammation, and wound healing [44]. NOS3 promotes the production of NO; NO can mediate vascular endothelial growth factor- (VEGF-) induced angiogenesis in coronary vessels and promote blood clotting through the activation of platelets [45].

### 3.6. H_H_-C_H_-T_H_ Network of QXH (Hemorheological Abnormality Module)

**Figure 3:**
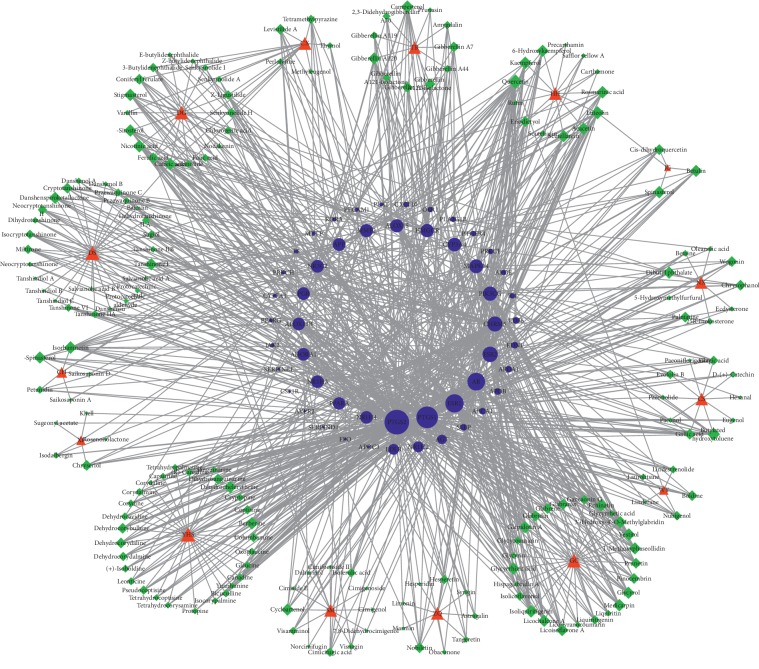
The H_H_-C_H_-T_H_ network of QXH (hemorheological abnormality module; red node: herbs, green node: compounds, and blue node: targets).

**Figure 4:**
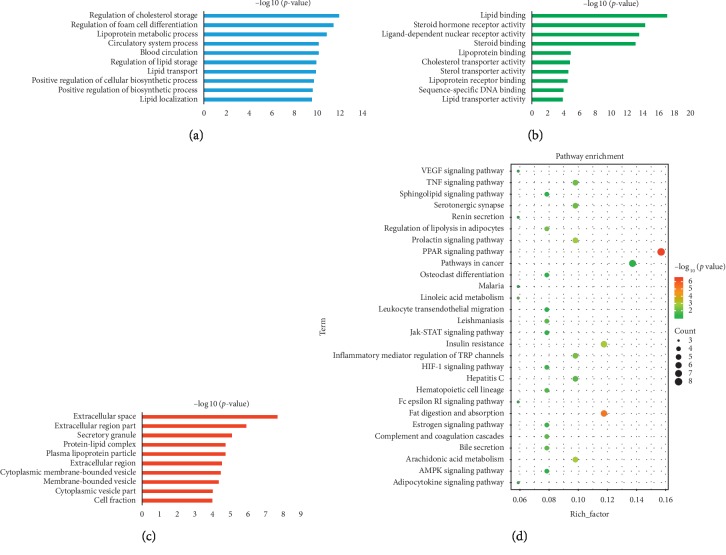
Gene ontology enrichment and KEGG pathway analysis of the targets of QXH related to hemorheological abnormality module. (a) Biological process. (b) Molecular function. (c) Cellular component. (d) KEGG pathway.

Hemorheological abnormality is an important part of the formation of QS-BSS, which is characterized by changes in blood fluidity, viscosity, deformability, and coagulability. Thus, we constructed a H_H_-C_H_-T_H_ network through network analysis to illuminate the relationship among herbs, active compounds, and candidate targets (Figure 3 (for original picture, see [Supplementary-material supplementary-material-1])). This network consists of 251 nodes (15 herbs, 185 active compounds, and 51 candidate targets; Tables [Supplementary-material supplementary-material-1] and [Supplementary-material supplementary-material-1]). As is depicted in the H_H_-C_H_-T_H_ network, red, green, and blue nodes represent herbs, active compounds, and candidate targets, respectively. The size of the nodes is positively related to the value of degree. Degree, which is the number of edges connected to the node, and the betweenness, another centrality index defined by the number of times a node acts a bridge along the shortest path between two other nodes, was calculated. Actually, betweenness reflects the fraction of the shortest paths in the network that pass through any particular node and a measure of the importance of a node as a hub in a network [46]. To gain insights into the pharmacological mechanisms of QXH on hemorheological abnormality, we performed gene ontology enrichment and KEGG pathway analysis on the 51 relevant targets, and the top 10 of biological process, molecular function, and cellular component and 28 KEGG pathways were predicted (Figure 4). The results of biological process were mainly enriched in regulation of cholesterol storage, lipoprotein metabolic process, blood circulation, and regulation of lipid storage, which were consistent with the pathologic mechanisms of hemorheological abnormality.

The degree and betweenness of the top 10 candidate compounds and targets involvied in hemorheological abnormality module are shown in Tables 4 and 5. Among the active compounds, quercetin, luteolin, isorhamnetin, butylated hydroxytoluene, and isoliquiritigenin were the top ones, interacting with 30, 13, 13, 12, and 12 targets, respectively. Quercetin acted on PTGS1, PTGS2, ALOX15, and PPARA and participated in the PPAR signaling pathway, prolactin signaling pathway, and VEGF signaling pathway. Numerous studies have shown that quercetin plays an important role in anti-inflammatory, antiproliferative, and antiatherosclerotic, especially in reducing blood fat and increasing coronary blood flow, which was contained by HH, TR, JG, WY, CH, and ZQ [40, 47]. Luteolin acting on PTGS1, PTGS2, AR, and APP could depress hypertensive aorta remodeling in spontaneous hypertensive rats, relax porcine coronary and aortic arteries, and improve cardiac function after myocardium ischemia/reperfusion injury [41, 42], which is vital to the treatment of cardiovascular diseases contained by HH, JG, XF, and ZQ. Butylated hydroxytoluene acting on PTGS1, PTGS2, AR, ESR1, and CYP3A4 is an antioxidant that has been shown to have an antiatherosclerotic effect in normal cholesterol-fed rabbits, which was contained by CS [48]. Kaempferol acting on AR, PIK3CG, ESR1, ESR2, CYP3A4, and APOE was involved in regulation of lipolysis in adipocytes, prolactin signaling pathway, estrogen signaling pathway, arachidonic acid metabolism, and VEGF signaling pathway, which is an important active member of dietary flavonoids discovered in many kinds of fruits and vegetables. Che et al. [49] demonstrated that kaempferol could effectively protect human umbilical vein endothelial cells against oxidative stress induced by ox-LDL, which indicated that kaempferol is capable of preventing atherosclerotic vascular disease. Rosmarinic acid acting on PTGS2, AR, ESR1, IL4, and NR1H4 was the active compound in DS and HH, which could inhibit the formation of malondialdehyde in human platelets *in vitro*, and its IC50 value is 3.37 nmol/L, indicating that rosmarinic acid has antiplatelet aggregation activity [50]. Berberine is a new cholesterol-lowering drug, and studies have shown that oral administration of berberine in 32 hypercholesterolemia patients for 3 months reduces serum cholesterol and triglycerides by 29% and 35%, respectively [51, 52].

Obviously, though the analysis of hemorheological abnormality module revealed that quercetin, luteolin, isorhamnetin, butylated hydroxytoluene, isoliquiritigenin, nicotinic acid, kaempferol, rosmarinic acid, campesterol, and berberine may play an important role in improving hemorheological abnormality, the hub targets of QXH in the treatment of QS-BSS were PTGS2, PTGS1, AR, ESR1, CHRM5, ESR2, MMP2, PIK3CG, CYP3A4, and HMGCR.

Quercetin was the active compound of HH, WY, CH, and ZQ; luteolin was the active compound of HH, JG, XF, and ZQ; isorhamnetin was only contained by CH; butylated hydroxytoluene was contained by CS; nicotinic acid was the active compound in DG; rosmarinic acid was contained by DS and HH; and campesterol and berberine were contained by TR and YHS, respectively. Kaempferol was the active compound of HH, CH, and ZQ. Based on this, we can infer that HH, CS, DG, TR, YHS, CH, and ZQ were exerting effects for improving hemorheological abnormality.

KEGG analysis showed that the targets (related to hemorheological abnormality) mainly involved in PPAR signaling pathway, fat digestion and absorption, arachidonic acid metabolism, prolactin signaling pathway, inflammatory mediator regulation of TRP channels, regulation of lipolysis in adipocytes, and TNF signaling pathway.

### 3.7. H_C_-C_C_-T_C_ Network of QXH (Coagulopathy Module)

**Figure 5:**
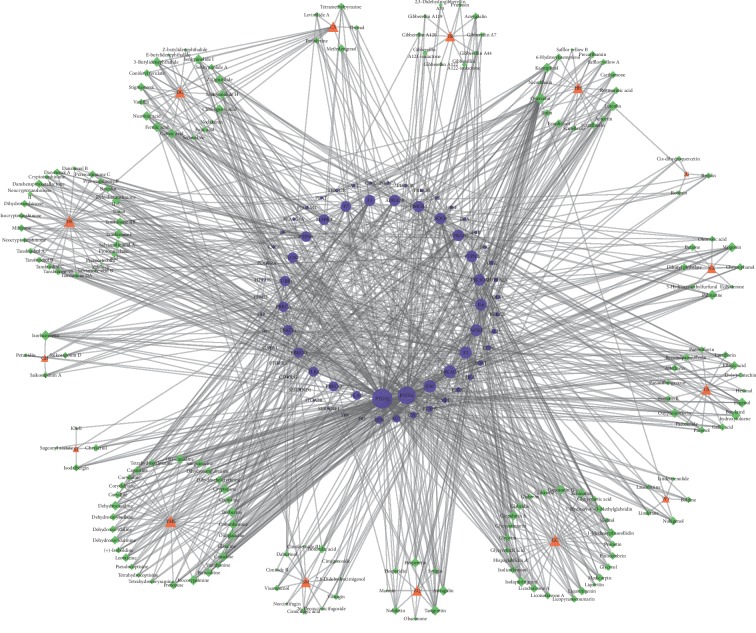
The H_C_-C_C_-T_C_ network of QXH (coagulopathy module, red node: herbs, green node: compounds, and blue node: targets).

Interestingly, quercetin, isorhamnetin, luteolin, kaempferol, berberine, wogonin, and isoliquiritigenin were not only playing a significant role in regulating hemorheological abnormality but also exerting effects for improving coagulopathy (Table 4). In Figure 5 (original picture see [Supplementary-material supplementary-material-1]), this network consisted of 267 nodes (15 herbs, 184 candidate compounds, and 68 candidate targets, Tables [Supplementary-material supplementary-material-1] and [Supplementary-material supplementary-material-1]). The degree and betweenness of the top 10 candidate compounds and targets involved in improving coagulopathy are shown in Tables 4 and 5. Tangeretin was one of the active compounds of ZQ, which interacted with the hub targets (such as PTGS1, PTGS2, IL6, F2, F10, NQO1, and HMOX1) in coagulation function module. Columbamine can significantly reduce the levels of TC, TG, and LDL-c in the serum of golden hamsters (HFHC) induced by high fat and high cholesterol, while increasing the level of HDL-c [53]. Ellagic acid was one of the main active compounds of CS, which acted on HMOX1, NQO1, F12, SYK, and NOS3, and was involved in PI3K-Akt signaling pathway and platelet activation. Tanshinone IIB acted on F2, PTGS2, MPI ,and other targets with antiplatelet aggregation activity [54]. Betaine was proved to protect against coagulation events in *vivo* and *vitro*, decreasing lipid peroxidation in plasma [55].

Remarkably, multiple molecular targets interacted with the bioactive compounds (Tables 4, and 5 and Figure 5), such as PTGS1, PTGS2, F10, PRKACA, F2, NOS3, IL6, PIK3CG, PTPN2, and ADRA2C. And 68 targets with relevant biological process demonstrated a closed relation to coagulation function. In platelets, PTGS1 and PTGS2 are involved in the generation of thromboxane A2, which promote platelet activation and aggregation, vasoconstriction, and proliferation of vascular smooth muscle cells [56].

Tangeretin was the active compound in ZQ. Ferulic acid was shared in DG, CX, and SM. Ellagic acid was only contained by CS. Columbamine was the active compound in YHS, and wogonin was contained by NX. Hence, we can infer that ZQ, CX, and SM also contributed to improving coagulopathy in addition to HH, DG, CS, TR, and YHS.

**Figure 6:**
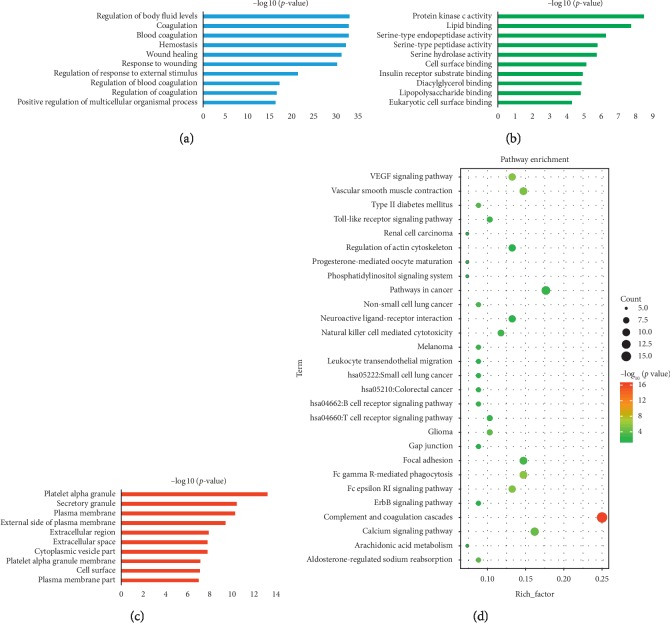
Gene ontology enrichment and KEGG pathway analysis of the targets of QXH related to coagulopathy module. (a) Biological process. (b) Molecular function. (c) Cellular component. (d) KEGG Pathway.

KEGG analysis (Figure 6) showed that the targets (related to coagulopathy) were mainly involved in complement and coagulation cascades, VEGF signaling pathway, vascular smooth muscle contraction, calcium signaling pathway, arachidonic acid metabolism, and leukocyte transendothelial migration. Over recent years, several studies have linked complement and thrombosis to the complement and coagulation cascades [57]. The thrombosis consists of insoluble fibrin, deposited platelets, accumulated white blood cells, and trapped red blood cells, which lead to the formation of blood stasis syndrome. VEGF signaling pathway leads to endothelial cell apoptosis, and a study has shown that the genetic repression of mouse VEGF expression regulates coagulation cascade [58]. Moreover, calcium signaling pathway plays a vital role in the development of platelet activation [59].

### 3.8. Analysis of the Overlapped Compounds, Targets, and Pathways in the Pathological Processes of Hemorheological Abnormality and Coagulopathy

**Figure 7:**
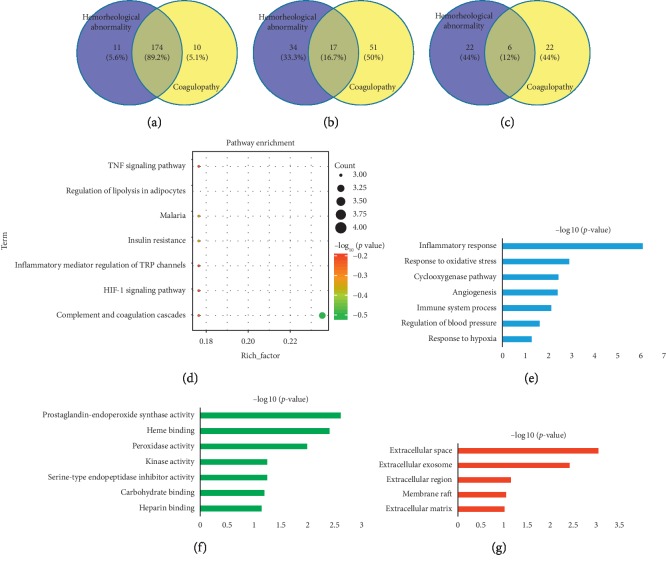
The analysis of overlapped compounds, targets, and pathways of the pathological processes: hemorheological abnormality and coagulopathy. (a) The overlapped compounds. (b) The overlapped targets. (c) The overlapped pathways. (d) KEGG pathway of the overlapped targets. (e) Biological process. (f) Molecular function. (g) Cellular component.

By analysis of the overlapped compounds, targets, and pathways involved in hemorheological abnormality and coagulopathy, 174 of 195 active compounds, 17 of 102 targets ([Supplementary-material supplementary-material-1]), and 6 of 50 pathways associated with QXH were shared in the improvement of hemorheological abnormality and coagulopathy, indicating QXH in treating QS-BSS by the synergy of multiple compounds, targets, and pathways (Figure 7). Then, the biological process of overlapped targets was analyzed, which indicated that the pathological processes of hemorheological abnormality and coagulopathy were involved in inflammatory response, response to oxidative stress, cyclooxygenase pathway, angiogenesis, immune system process, regulation of blood pressure, and response to hypoxia of total 6 biological processes. Hence, in addition to hemorheological abnormality and coagulopathy, the participation of inflammation and immunity in the formation of QS-BSS is also worthy of our attention. Complement and coagulation cascades, arachidonic acid metabolism, VEGF signaling pathway, leukocyte transendothelial migration, Fc epsilon RI signaling pathway, and pathways in cancer might be the hub pathways of QXH in treating QS-BSS, which were shared in improving hemorheological abnormality and coagulopathy.

## 4. Discussion

TCM is a precious heritage which has been practiced for thousands of years in China and surrounding countries. Nowadays, herbal medicine has been widely used for complicated disease treatment and is gradually becoming acceptable as an alternative medicine worldwide [60]. TCM network pharmacology was a new research strategy for translating TCM from an experience-based medicine to an evidence-based medicine system, which predicted the target profiles and pharmacological actions of herbal compounds and revealed drug-gene-disease comodule associations to interpret the combinatorial rules and network regulation effects of herbal formula [61]. Therefore, we herein utilized a combination method that included pharmacodynamics evaluation and network pharmacology to elucidate the pharmacodynamic effect, substances, and potential mechanisms of QXH through improving hemorheology and coagulation function in treating QS-BSS.

Hemorheology and coagulation function provide an important approach for studies on the mechanisms of QS-BSS and the therapeutic effects of Chinese herbal compounds that promote blood circulation and remove blood stasis [16]. In our work, we established the QS-BSS model in rats to evaluate the pharmacodynamic effect of QXH in improving hemorheological abnormality and coagulopathy. CDDP, one Chinese patent drug, was used as the positive control drug in this study. Preclinical and clinical studies have suggested that CDDP can expand blood vessel, promote blood circulation, relieve blood stasis, improve microcirculation, and improve hemorheological property [62]. The results of pharmacodynamic evaluation showed that QXH and CDDP groups significantly regulated the hemorheological abnormality and coagulopathy in rats with QS-BSS. Especially, QXH-L and QXH-H groups could significantly improve the abnormal increase of WBV and PV in rats with QS-BSS and had different degrees of callback on APTT, TT, PT, and FIB, which are comparable with CDDP in improving hemorheology and coagulation function. In terms of PCA and PLS-DA, after the preadministration of QXH, the same parameters in the QS-BSS model were close to those in the control and CDDP groups, also indicating that QXH has significant pharmacodynamic effect on QS-BSS. All of the above results were consistent with those of previous studies [63–65]. Hence, QXH, through promoting blood circulation and removing blood stasis in treating QS-BSS, gives us a glance at TCM compound formula in the treatment of the disease with a variety of pathogenesis by the synergy of various herbal medicines.

Thereby, we performed a network pharmacology approach which contains OB, Caco-2, and DL screening, active compound verification, target prediction, and network analysis to dissect the active compounds and possible mechanisms of QXH on regulating hemorheological abnormality and coagulopathy. The main results are as follows: (1) 195 active compounds in 15 herbs and 102 targets were identified as candidate targets related to QS-BSS. These active compounds provided clues to our study of the substance on the basis of the pharmacodynamic effect of QXH. (2) Meanwhile, 25 active compounds were verified by UPLC-Q/TOF-MS on QXH methanol and water extracts, which are corresponding to the results of network pharmacology. (3) Through the network analysis of QXH on the two vital pathological processes of QS-BSS, the mechanism of QXH in the treatment of QS-BSS is gradually clear.

In the hemorheological abnormality network module, 51 targets participated in signaling pathways such as PPAR signaling pathway, prolactin signaling pathway, fat digestion and absorption, estrogen signaling pathway, VEGF signaling pathway, and leukocyte transendothelial migration. A recent study on antihyperlipidemic activity of quercetin (included in HH, NX, WY, YHS, CH, XF, and ZQ) revealed that it may be involved in regulating the PPAR signaling pathway [66], which is comparable to our findings. The PPAR signal pathway is concerned with the formation of QS-BSS. PPAR*γ* is highly expressed in macrophage-derived foam cells of both early and advanced atherosclerotic lesions [67]. PPAR*γ* ligands may attenuate inflammation and hence, atherosclerosis in the vessel wall. But the PPAR*γ* expression in monocytes increased the expression of CD36, leading to an enhanced macrophage uptake of oxidized LDL, thus enhancing foam cell formation [68].

In the coagulopathy network module, 68 targets participated in signaling pathways such as complement and coagulation cascades, VEGF signaling pathway, vascular smooth muscle contraction, calcium signaling pathway, arachidonic acid metabolism, and long-term depression. F2, F3, F7, F9, F10, SERPINE1, and SERPIND1 were included by complement and coagulation cascades, which acted on blood coagulation, platelet activation, blood homeostasis, inflammation, and wound healing. Studies have shown that APTT and PT reflect changes in the coagulation factor of the endogenous and exogenous coagulation pathways, respectively. TT and FIB are related to the common coagulation pathway in plasma [36]. However, the analysis results of coagulopathy network module fully support the rationality of using APTT, TT, PT, and FIB to evaluate QXH on improving coagulation function in QS-BSS. Another study on identifying the compounds of DS on intervening thrombotic diseases revealed that tanshinone IIB could improvemicrocirculation and prevent thrombosis involved in complement and coagulation cascades based on UPLC-LTQ-Orbitrap MS [69], which is also comparable to our findings.

Analysis of the targets overlapped such as PTGS1, PTGS2, PIK3CG, and PECAM1 in the pathological processes of hemorheological abnormality and coagulopathy implied that inflammation and immunity were also important for QS-BSS which need further study. Complement and coagulation cascades, arachidonic acid metabolism, and VEGF signaling pathway were shared in hemorheological abnormality and coagulopathy, which need more attention, and further experimental validation will be carried out.

However, although we have predicted the main active compounds of QXH through network pharmacology and some of which has been verified by UPLC-Q/TOF-MS, to clarify the substance basis of the pharmacodynamic effect of QXH, further study on the migrating components absorbed into blood by serum pharmacochemistry based on animal experiments is needed [70]. Meanwhile, the network analysis also provided a vital reference for experimental investigations of hub targets and signaling pathways of QXH in treating QS-BSS.

## 5. Conclusions

Through the pharmacodynamic evaluation of QXH based on animal experiments and the underlying pharmacological mechanism prediction of QXH, our study has proved that the effect of QXH in the treatment of QS-BSS is significant, and the underlying pharmacological mechanisms might be related to complement and coagulation cascades, leukocyte transendothelial migration, PPAR signaling pathway, VEGF signaling pathway, arachidonic acid metabolism, inflammatory mediator regulation of TRP channels, and TNF signaling pathway, for which the further experimental validation is essential.

## Figures and Tables

**Table 1:** Latin names, the number of active compounds of each herb in QXH involving in mediating hemorheological abnormality and coagulopathy, and the corresponding abbreviations of each herb.

No.	Name	Active compounds	Number		Abbreviations
			Hemorheological abnormality	Coagulopathy	
1	Angelicae Sinensis Radix	19	18	17	DG
2	Paeoniae Radix Rubra	17	10	15	CS
3	Chuanxiong Rhizoma	15	15	15	CX
4	Persicae Semen	12	11	10	TR
5	Carthami Flos	15	13	15	HH
6	Bupleuri Radix	11	7	6	CH
7	Cyperi Rhizoma	11	6	5	XF
8	Salviae Miltiorrhizae Radix et Rhizoma	29	28	27	DS
9	Corydalis Rhizoma	33	30	30	YHS
10	Platycodonis Radix	6	4	4	JG
11	Aurantii Fructus	14	13	12	ZQ
12	Linderae Radix	7	6	6	WY
13	Achyranthis Bidentatae Radix	13	9	8	NX
14	Cimicifugae Rhizoma	19	14	13	SM
15	Glycyrrhizae Radix et Rhizoma	26	25	25	GC

**Table 2:** Effects of QXH on hemorheology in rats with QS-BSS ($\bar{x}\pm s$, *n* = 6∼8).

Group	Doses	WBV (MPa × s)					PV
	g × kg^−1^	200 s^−1^	100 s^−1^	50 s^−1^	5 s^−1^	1 s^−1^	
Control	—	3.63 ± 0.10	4.01 ± 0.15	4.46 ± 0.20	8.66 ± 0.50	19.44 ± 1.39	1.24 ± 0.07
Model	—	4.22 ± 0.14^++^	4.70 ± 0.23^++^	5.44 ± 0.29^++^	14.76 ± 1.44^++^	33.61 ± 3.19^++^	2.17 ± 0.43^++^
QXH-L	0.432	3.77 ± 0.17^*∗∗*^	4.07 ± 0.18^*∗∗*^	4.49 ± 0.21^*∗∗*^	8.29 ± 0.48^*∗∗*^	18.07 ± 1.34^*∗∗*^	1.33 ± 0.15^*∗∗*^
QXH-M	1.296	3.93 ± 0.10^*∗*^	4.32 ± 012^*∗∗*^	4.88 ± 0.17^*∗∗*^	10.11 ± 0.83^*∗∗*^	24.39 ± 3.03^*∗∗*^	1.33 ± 0.15^*∗∗*^
QXH-H	2.592	3.74 ± 0.15^*∗∗*^	4.06 ± 0.18^*∗∗*^	4.54 ± 0.22^*∗∗*^	8.92 ± 0.91^*∗∗*^	20.60 ± 3.24^*∗∗*^	1.42 ± 0.08^*∗∗*^
CDDP	0.1	3.84 ± 0.22^*∗∗*^	4.10 ± 0.23^*∗∗*^	4.64 ± 0.38^*∗∗*^	8.39 ± 0.69^*∗∗*^	17.94 ± 1.62^*∗∗*^	1.7 ± 0.28^*∗∗*^

WBV: whole blood viscosity; PV: plasma viscosity; 200 s^−1^, 100 s^−1^, 50 s^−1^, 5 s^−1^, and 1 s^−1^ represent whole blood viscosity in 200 s^−1^, 100 s^−1^, 50 s^−1^, 5 s^−1^, and 1 s^−1^ shear rates, respectively. Compared with the control group, ^+^*p* < 0.05 and ^++^*p* < 0.01; compared with the model group, ^*∗*^*p* < 0.05 and ^*∗∗*^*p* < 0.01.

**Table 3:** Effects of QXH on coagulation function in rats with QS-BSS ($\bar{x}\pm s$, *n* = 6∼8).

Group	Doses	Coagulation function			
	g × kg^−1^	TT (s)	PT (s)	APTT (s)	FIB (g/L)
Control	—	25.34 ± 1.14	12.21 ± 1.17	21.45 ± 2.10	2.94 ± 0.40
Model	—	21.70 ± 1.51^+^	9.25 ± 0.38^++^	16.64 ± 0.96^++^	4.81 ± 0.47^++^
QXH-L	0.432	25.21 ± 1.69^*∗*^	12.03 ± 2.28^*∗*^	18.24 ± 2.66	3.93 ± 0.81
QXH-M	1.296	27.98 ± 0.56^*∗∗*^	9.96 ± 0.847	21.78 ± 3.10^*∗*^	4.12 ± 0.64
QXH-H	2.592	28.20 ± 1.29^*∗∗*^	11.22 ± 1.25	19.05 ± 1.90	4.30 ± 0.73
CDDP	0.1	24.36 ± 3.74^*∗∗*^	11.04 ± 1.35	25.5 ± 1.33^*∗∗*^	3.64 ± 0.50^*∗*^

TT: thrombin time; PT: prothrombin time; APTT: activated partial thromboplastin; FIB: fibrinogen content. Compared with the control group, ^+^*p* < 0.05 and ^++^*p* < 0.01; compared with the model group ^*∗*^*p* < 0.05 and ^*∗∗*^*p* < 0.01.

**Table 4:** Top 10 candidate compounds (hemorheological abnormality module and coagulopathy module) according to 2 centrality indexes.

Compounds	Degree	Compounds	Betweenness
*Hemorheological abnormality*			
Quercetin	32	Quercetin	0.0857877
Luteolin	16	Luteolin	0.02594642
Isorhamnetin	13	Butylated hydroxytoluene^*∗*^	0.01562293
Butylated hydroxytoluene^*∗*^	12	Isorhamnetin	0.01488112
Isoliquiritigenin	12	Isoliquiritigenin^*∗*^	0.01377775
Nicotinic acid	12	Gallic acid	0.01187844
Kaempferol	12	Berberine^*∗*^	0.01178984
Rosmarinic acid^*∗*^	11	Nicotinic acid	0.01170317
Campesterol	11	Glycyrrhizic acid^*∗*^	0.01089196
Berberine^*∗*^	10	Rosmarinic acid^*∗*^	0.01077513
*Coagulopathy*			
Quercetin	34	Quercetin	0.0827979
Isorhamnetin	16	Luteolin	0.0211428
Luteolin	14	Wogonin	0.0211385
Kaempferol	14	Isorhamnetin	0.020552
Tangeretin^*∗*^	13	Ellagic acid	0.0161838
Berberine^*∗*^	13	Tanshinone IIB	0.0145945
Columbamine	11	Betaine	0.0142141
Wogonin	11	Berberine^*∗*^	0.0130752
Z-ligustilide^*∗*^	10	Ferulic acid^*∗*^	0.0130271
Ferulic acid^*∗*^	10	Tangeretin^*∗*^	0.0124719

The active compounds which had been verified by UPLC-Q/TOF-MS in QXH were labeled with ^*∗*^.

**Table 5:** Top 10 candidate targets (hemorheological abnormality module and coagulopathy module) according to 2 centrality indexes.

Targets	Degree	Targets	Betweenness
*Hemorheological abnormality*			
PTGS2	149	PTGS2	0.39630531
PTGS1	115	PTGS1	0.1906698
ESR1	77	AR	0.09648379
AR	70	ESR1	0.09300286
ESR2	43	CHRM5	0.03405704
CHRM5	39	ESR2	0.02949908
PIK3CG	29	MMP2	0.02707469
MAPK14	25	PIK3CG	0.01870076
CYP3A4	23	CYP3A4	0.0143101
HMGCR	19	HMGCR	0.01310125
*Coagulopathy*			
PTGS2	149	PTGS2	0.4147934
PTGS1	115	PTGS1	0.1977836
F10	45	IL6	0.0557075
PRKACA	45	F2	0.0542773
F2	40	F10	0.0379244
NOS3	34	NOS3	0.0275241
IL6	33	PRKACA	0.0275035
PIK3CG	29	PTPN2	0.0243642
PTPN2	28	NQO1	0.0201226
ADRA2C	28	HMOX1	0.0175608

## Data Availability

The data that support the findings of this study are available in the Supplementary Materials and openly available in the PubChem (https://pubchem.ncbi.nlm.nih.gov), ChemSpider (http://www.chemspider.com), TCMSP (http://lsp.nwu.edu.cn/index.php), TCM-MESH (http://mesh.tcm.microbioinformatics.org), STITCH (http://stitch.embl.de/), Therapeutic Targets Database (TTD, http://bidd.nus.edu.sg/group/ttd/), Comparative Toxicogenomics Database (CTD, http://ctdbase.org/), the Similarity Ensemble Approach (SEA, http://sea.bkslab.org/), Swiss Target Prediction webserver (http://www.swisstargetprediction.ch), Online Mendelian Inheritance in Man Database (OMIM, http://www.omim.org/), and Uniprot (https://www.uniprot.org).

## Abbreviations

QXH: Qixuehe capsule
QXH-L: Low-dose QXH-intervened
QXH-M: Middle-dose QXH-intervened
QXH-H: High-dose QXH-intervened
TCM: Traditional Chinese medicine
Adr: Adrenaline hydrochloride
CDDP: Compound danshen dripping pills
PT: Prothrombin time
APTT: Activated partial thromboplastin time
TT: Thrombin time
FIB: Fibrinogen content
WBV: Whole blood viscosity
PV: Plasma viscosity
PCA: Principal component analysis
PLS: Partial least squares
CV-ANOVA: Analysis of variance testing of cross-validated predictive residuals
UPLC: Ultra-performance liquid chromatography
MS: Mass spectrometer
TCMSP: Traditional Chinese medicine systems pharmacology database and analysis platform
TTD: Therapeutic targets database
CTD: Comparative toxicogenomics database
SEA: Similarity ensemble approach
OMIM: Online Mendelian inheritance in man database
OB: Oral bioavailability
Caco-2: Caco-cell permeability
DL: Drug-likeness
KEGG: Kyoto Encyclopedia of Genes and Genomes
DAVID: Database for Annotation, Visualization and Integrated Discovery
DG: Angelicae Sinensis Radix
CS: Paeoniae Radix Rubra
CX: Chuanxiong Rhizoma
TR: Persicae Semen
HH: Carthami Flos
CH: Bupleuri Radix
XF: Cyperi Rhizoma
DS: Salviae Miltiorrhizae Radix et Rhizoma
YHS: Corydalis Rhizoma
JG: Platycodonis Radix
ZQ: Aurantii Fructus
WY: Linderae Radix
NX: Achyranthis Bidentatae Radix
SM: Cimicifugae Rhizoma
GC: Glycyrrhizae Radix et Rhizoma.

## Supplementary Materials

Supplementary Materials Table S1: the OB, DL, and Caco-2 of the active compounds in QXH. Table S2: the active compounds involved in hemorheological abnormality module and coagulopathy module. Table S3: the candidate targets involved in hemorheological abnormality module and coagulopathy module. Table S4: the overlapped targets in hemorheological abnormality module and coagulopathy module. Figure S1: PCA score plots of hemorheology and coagulation function indexes among all groups. Figure S2: the permutation tests (*n* = 200) (A) and CV-AVNOA tests (B) for the PLS-DA model among all groups. Figure S3: PLS-DA score plots of hemorheology and coagulation function indexes between control and model groups. Figure S4: the permutation tests (*n* = 200) (A) and CV-AVNOA tests (B) for the PLS-DA model between control and model groups. Figure S5: base peak intensity chromatograms of 25 standards in positive ion mode or negative ion mode. Figure S6: base peak intensity chromatograms of QXH methanol extracts (A) in positive ion mode and (C) in negative ion mode; base peak intensity chromatograms of QXH water extracts (B) in positive ion mode and (D) in negative ion mode by UPLC-Q/TOF-MS. Figure S7: the HH-CH-TH network of QXH (hemorheological abnormality module; red node: herbs, green node: compounds, and blue node: targets) (the original picture for Figure 3). Figure S8: the HC-CC-TC network of QXH (coagulopathy module, red node: herbs, green node: compounds, and blue node: targets) (the original picture for Figure 5).
