# Supplementary material for: Deciphering the Active Compounds and Mechanisms of Qixuehe Capsule on Qi Stagnation and Blood Stasis Syndrome: A Network Pharmacology Study
Source: Evid Based Complement Alternat Med. 2020 Feb 27;2020:5053914. doi: 10.1155/2020/5053914 (PMC7063220; doi:10.1155/2020/5053914)
Supplement: Supplementary Materials — Table S1: the OB, DL, and Caco-2 of the active compounds in QXH. Table S2: the active compounds involved in hemorheological abnormality module and coagulopathy module. Table S3: the candidate targets involved in hemorheological abnormality module and coagulopathy module. Table S4: the overlapped targets in hemorheological abnormality module and coagulopathy module. Figure S1: PCA score plots of hemorheology and coagulation function indexes among all groups. Figure S2: the permutation tests (n = 200) (A) and CV-AVNOA tests (B) for the PLS-DA model among all groups. Figure S3: PLS-DA score plots of hemorheology and coagulation function indexes between control and model groups. Figure S4: the permutation tests (n = 200) (A) and CV-AVNOA tests (B) for the PLS-DA model between control and model groups. Figure S5: base peak intensity chromatograms of 25 standards in positive ion mode or negative ion mode. Figure S6: base peak intensity chromatograms of QXH methanol extracts (A) in positive ion mode and (C) in negative ion mode; base peak intensity chromatograms of QXH water extracts (B) in positive ion mode and (D) in negative ion mode by UPLC-Q/TOF-MS. Figure S7: the HH-CH-TH network of QXH (hemorheological abnormality module; red node: herbs, green node: compounds, and blue node: targets) (the original picture for Figure 3). Figure S8: the HC-CC-TC network of QXH (coagulopathy module, red node: herbs, green node: compounds, and blue node: targets) (the original picture for Figure 5). [file 5053914.f1.docx]

**Table S1: The OB, DL, and Caco-2 of the active compounds in QXH.**

| **Herbs** | **Active compounds** | **OB (%)** | **DL** | **Caco-2** | **screening criteria** |
| --- | --- | --- | --- | --- | --- |
| DG | Z-Ligustilide* | 51.3 | 0.07 | 1.31 | failed to pass |
| DG | Senkyunolide A | 26.56 | 0.07 | 1.3 | failed to pass |
| DG | Senkyunolide I | 46.8 | 0.08 | 1 | failed to pass |
| DG | Senkyunolide K | 61.75 | 0.08 | 0.52 | failed to pass |
| DG | Z-butylidenephthalide | 53.72 | 0.07 | 1.3 | failed to pass |
| DG | E-butylidenephthalide | 42.44 | 0.07 | 1.32 | failed to pass |
| DG | 3-Butylidenephthalide | 42.44 | 0.07 | 1.32 | failed to pass |
| DG | Coniferyl ferulate | 4.54 | 0.39 | 0.71 | failed to pass |
| DG | Stigmasterol* | 43.83 | 0.76 | 1.44 | pass |
| DG | Vanillin | 52 | 0.03 | 0.68 | failed to pass |
| DG | β-Sitosterol* | 36.91 | 0.75 | 1.32 | pass |
| DG | Nicotinic acid | 47.65 | 0.02 | 0.34 | failed to pass |
| DG | Ferulic acid* | 39.56 | 0.06 | 0.47 | failed to pass |
| DG | Caffeic acid* | 54.97 | 0.05 | 0.27 | failed to pass |
| DG | Sedanolide | 62.46 | 0.07 | 1.24 | failed to pass |
| DG | Folic acid | 68.96 | 0.71 | -1.5 | failed to pass |
| DG | Nodakenin | 57.12 | 0.69 | -0.79 | failed to pass |
| DG | Chlorogenic acid | 11.93 | 0.33 | -1.03 | failed to pass |
| DG | Senkyunolide H | 41.04 | 0.09 | 0.91 | failed to pass |
| CS | β-Sitosterol* | 36.91 | 0.75 | 1.32 | pass |
| CS | D-(+)-Catechin | 54.83 | 0.24 | -0.03 | pass |
| CS | Ellagic acid | 43.06 | 0.43 | -0.44 | failed to pass |
| CS | Lactiflorin* | 49.12 | 0.8 | -1.13 | failed to pass |
| CS | Paeoniflorin | 53.87 | 0.79 | -1.47 | failed to pass |
| CS | Benzoylpaeoniflorin* | 31.14 | 0.54 | -0.85 | failed to pass |
| CS | Albiflorin* | 30.25 | 0.77 | -1.52 | failed to pass |
| CS | Paeoniflorigenone | 65.33 | 0.37 | -0.13 | pass |
| CS | Evofolin B | 64.74 | 0.22 | 0 | pass |
| CS | Paeonioflorin* | 10.22 | 0.79 | -1.69 | failed to pass |
| CS | Oxypaeoniflorin* | 8.38 | 0.78 | -1.62 | failed to pass |
| CS | Paeonolide | 6.3 | 0.64 | -1.42 | failed to pass |
| CS | Paeonol | 28.79 | 0.04 | 0.93 | failed to pass |
| CS | Gallic acid | 31.69 | 0.04 | -0.09 | failed to pass |
| CS | Butylated hydroxytoluene* | 40.02 | 0.07 | 1.75 | failed to pass |
| CS | Eugenol | 56.24 | 0.04 | 1.35 | failed to pass |
| CS | Hexanal | 55.71 | 0.01 | 1.25 | failed to pass |
| CX | Ferulic acid* | 39.56 | 0.06 | 0.47 | failed to pass |
| CX | Caffeic acid* | 54.97 | 0.05 | 0.27 | failed to pass |
| CX | Coniferyl ferulate | 4.54 | 0.39 | 0.71 | failed to pass |
| CX | Z-Ligustilide* | 51.3 | 0.07 | 1.31 | failed to pass |
| CX | 3-Butylidenephthalide | 42.44 | 0.07 | 1.32 | failed to pass |
| CX | Senkyunolide A | 26.56 | 0.07 | 1.3 | failed to pass |
| CX | Senkyunolide I | 46.8 | 0.08 | 1 | failed to pass |
| CX | Senkyunolide H | 41.04 | 0.09 | 0.91 | failed to pass |
| CX | Tetramethylpyrazine | 20.01 | 0.03 | 1.19 | failed to pass |
| CX | Levistilide A* | 16.56 | 0.05 | 0.83 | failed to pass |
| CX | Vanillin | 52 | 0.03 | 0.68 | failed to pass |
| CX | Perlolyrine | 65.95 | 0.27 | 0.88 | pass |
| CX | Methyleugenol | 73.36 | 0.04 | 1.47 | failed to pass |
| CX | Sedanolide | 62.46 | 0.07 | 1.24 | failed to pass |
| CX | Thymol | 41.47 | 0.03 | 1.6 | failed to pass |
| TR | Amygdalin* | 4.42 | 0.61 | -1.91 | failed to pass |
| TR | Prunasin | 12.61 | 0.18 | -0.79 | failed to pass |
| TR | β-Sitosterol* | 36.91 | 0.75 | 1.32 | pass |
| TR | Campesterol | 37.58 | 0.71 | 1.31 | pass |
| TR | 2,3-Didehydrogibberellin A70 | / | / | / | failed to pass |
| TR | Gibberellin A119 | / | / | / | failed to pass |
| TR | Gibberellin A120 | / | / | / | failed to pass |
| TR | Gibberellin A121-isolactone | / | / | / | failed to pass |
| TR | Gibberellin A122 | / | / | / | failed to pass |
| TR | Gibberellin A122-isolactone | / | / | / | failed to pass |
| TR | Gibberellin A44 | 101.61 | 0.54 | -0.13 | pass |
| TR | Gibberellin A7 | / | / | / | failed to pass |
| JG | Acacetin | 34.97 | 0.24 | 0.67 | pass |
| JG | Luteolin | 36.16 | 0.25 | 0.19 | pass |
| JG | Cis-dihydroquercetin | 66.44 | 0.27 | -0.34 | pass |
| JG | Spinasterol | 42.98 | 0.76 | 1.44 | pass |
| JG | Robinin | 39.84 | 0.71 | -1.48 | failed to pass |
| JG | Betulin | 15.48 | 0.78 | 0.84 | failed to pass |
| NX | Quercetin | 46.43 | 0.28 | 0.05 | pass |
| NX | Wogonin | 30.68 | 0.23 | 0.79 | pass |
| NX | Oleanolic acid | 29.02 | 0.76 | 0.59 | failed to pass |
| NX | kaempferol | 41.88 | 0.24 | 0.26 | pass |
| NX | Betaine | 40.92 | 0.01 | -0.77 | failed to pass |
| NX | Stigmasterol* | 43.83 | 0.76 | 1.44 | pass |
| NX | Dibutyl phthalate | 64.54 | 0.13 | 0.8 | failed to pass |
| NX | 5-Hydroxymethylfurfural | 45.07 | 0.02 | 0.05 | failed to pass |
| NX | Palmatine | 64.6 | 0.65 | 1.33 | pass |
| NX | 25R-Inokosterone | 9.03 | 0.83 | -1.52 | failed to pass |
| NX | β-Sitosterol* | 36.91 | 0.75 | 1.32 | pass |
| NX | Ecdysterone | 44.23 | 0.82 | -1.36 | failed to pass |
| NX | Chrysophanol | 18.64 | 0.21 | 0.62 | failed to pass |
| WY | Lindestrenolide | / | / | / | failed to pass |
| WY | Laurolitsine | 40.92 | 0.46 | 0.75 | pass |
| WY | Linderane | / | / | / | failed to pass |
| WY | Nubigenol | 42.55 | 0.19 | -0.21 | pass |
| WY | Boldine | 31.18 | 0.51 | 1.05 | pass |
| WY | β-Sitosterol* | 36.91 | 0.75 | 1.32 | pass |
| WY | Quercetin | 46.43 | 0.28 | 0.05 | pass |
| DS | Luteolin | 36.16 | 0.25 | 0.19 | pass |
| DS | Sugiol | 36.11 | 0.28 | 1.14 | pass |
| DS | Dehydrotanshinone II A | 43.76 | 0.4 | 1.02 | pass |
| DS | Baicalin | 40.12 | 0.75 | -0.85 | failed to pass |
| DS | Przewaquinone B | 62.24 | 0.41 | 0.39 | pass |
| DS | Przewaquinone C | 55.74 | 0.4 | 0.42 | pass |
| DS | Danshenol B | 57.95 | 0.56 | 0.53 | pass |
| DS | Danshenol A | 56.97 | 0.52 | 0.33 | pass |
| DS | Cryptotanshinone* | 52.34 | 0.4 | 0.95 | pass |
| DS | Danshenspiroketallactone | 50.43 | 0.31 | 0.88 | pass |
| DS | Neocryptotanshinone II | 39.46 | 0.23 | 0.76 | pass |
| DS | Dihydrotanshinone I | 45.04 | 0.36 | 0.95 | pass |
| DS | Isocryptotanshinone | 17.99 | 0.44 | 0.48 | failed to pass |
| DS | Miltirone | 38.76 | 0.25 | 1.23 | pass |
| DS | Neocryptotanshinone | 52.49 | 0.32 | 0.35 | pass |
| DS | Tanshindiol A | / | / | / | failed to pass |
| DS | Tanshindiol B | 42.67 | 0.45 | 0.05 | pass |
| DS | Tanshindiol C | / | / | / | failed to pass |
| DS | Tanshinone VI | 45.64 | 0.3 | 0.48 | pass |
| DS | Tanshinone IIA* | 49.89 | 0.4 | 1.05 | pass |
| DS | Salvianolic acid B | 3.01 | 0.41 | -1.67 | failed to pass |
| DS | Danshensu | / | / | / | failed to pass |
| DS | Protocatechuic aldehyde | 38.35 | 0.03 | 0.43 | failed to pass |
| DS | Protocatechuic acid | 25.37 | 0.04 | 0.1 | failed to pass |
| DS | Caffeic acid* | 54.97 | 0.05 | 0.27 | failed to pass |
| DS | Rosmarinic acid* | 1.38 | 0.35 | -0.54 | failed to pass |
| DS | Salvianolic acid A | 2.96 | 0.7 | -0.56 | failed to pass |
| DS | Tanshinone I* | 29.27 | 0.36 | 1.05 | failed to pass |
| DS | Tanshinone IIB | 21.07 | 0.45 | 0.38 | failed to pass |
| YHS | Berberine* | 36.86 | 0.78 | 1.24 | pass |
| YHS | Coptisine | 30.67 | 0.86 | 1.21 | pass |
| YHS | Cryptopine | 25.53 | 0.72 | 0.86 | failed to pass |
| YHS | Dihydrochelerythrine | 32.73 | 0.81 | 1.13 | pass |
| YHS | Dihydrosanguinarine | 59.31 | 0.86 | 1 | pass |
| YHS | Sanguinarine | 37.81 | 0.86 | 1.26 | pass |
| YHS | (R)-Canadine | 55.37 | 0.77 | 1.04 | pass |
| YHS | β-Sitosterol* | 36.91 | 0.75 | 1.32 | pass |
| YHS | Tetrahydropalmatine* | 73.94 | 0.64 | 1 | pass |
| YHS | Capaurine | 62.91 | 0.69 | 0.86 | pass |
| YHS | Corydaline | 65.84 | 0.68 | 1.23 | pass |
| YHS | Corydalmine | 52.5 | 0.59 | 1.21 | pass |
| YHS | Corydine | 37.16 | 0.55 | 1.29 | pass |
| YHS | Dehydrocavidine | 38.99 | 0.81 | 1.21 | pass |
| YHS | Dehydrocorybulbine | 46.97 | 0.63 | 1.32 | pass |
| YHS | Dehydrocorydaline | 41.98 | 0.68 | 1.35 | pass |
| YHS | Dehydrocorydalmine | 43.9 | 0.59 | 1.2 | pass |
| YHS | (+)-Isoboldine | 39.53 | 0.51 | 0.84 | pass |
| YHS | Leonticine | 45.79 | 0.26 | 1.21 | pass |
| YHS | Pseudocoptisine | 38.97 | 0.86 | 1.23 | pass |
| YHS | Tetrahydrocoptisine | 48.25 | 0.85 | 0.93 | pass |
| YHS | Tetrahydrocorysamine | 34.17 | 0.86 | 1.07 | pass |
| YHS | Stigmasterol* | 43.83 | 0.76 | 1.44 | pass |
| YHS | Palmatine | 64.6 | 0.65 | 1.33 | pass |
| YHS | Protopine | / | / | / | failed to pass |
| YHS | Isocorypalmine | 35.77 | 0.59 | 0.85 | pass |
| YHS | Bicuculline | 69.67 | 0.88 | 0.72 | pass |
| YHS | Quercetin | 46.43 | 0.28 | 0.05 | pass |
| YHS | Yuanhunine | / | / | / | failed to pass |
| YHS | Canadine | 55.37 | 0.77 | 1.04 | pass |
| YHS | Glaucine | 29.03 | 0.61 | 1.35 | failed to pass |
| YHS | Oxoglaucine | 26.61 | 0.62 | 0.69 | failed to pass |
| YHS | Columbamine | 26.94 | 0.59 | 1.01 | failed to pass |
| SM | Cimigenoside | / | / | / | failed to pass |
| SM | Isoferulic acid* | 50.83 | 0.06 | 0.49 | failed to pass |
| SM | Cimifoetiside III | / | / | / | failed to pass |
| SM | Dahurinol | 17.32 | 0.6 | -0.01 | failed to pass |
| SM | Cimiside E | 16.49 | -0.3 | 0.16 | failed to pass |
| SM | Cycloartenol | 38.69 | 0.78 | 1.53 | pass |
| SM | Caffeic acid* | 54.97 | 0.05 | 0.27 | failed to pass |
| SM | β-Sitosterol* | 36.91 | 0.75 | 1.32 | pass |
| SM | Cimiside B | / | / | / | failed to pass |
| SM | Visamminol | 50.01 | 0.23 | 0.41 | pass |
| SM | Norcimifugin | / | / | / | failed to pass |
| SM | Cimicifugic acid | 83.02 | 0.45 | -0.11 | pass |
| SM | 26-Deoxycimicifugoside | / | / | / | failed to pass |
| SM | Shengmanol xyloside | / | / | / | failed to pass |
| SM | Visnagin | 44.25 | 0.15 | 1.1 | failed to pass |
| SM | Ferulic acid* | 39.56 | 0.06 | 0.47 | failed to pass |
| SM | Cimicifugin | / | / | / | failed to pass |
| SM | 7,8-Didehydrocimigenol | 36.79 | 0.4 | 0.14 | pass |
| SM | Cimigenol | 37.19 | 0.4 | -0.16 | pass |
| GC | 1-Methoxyphaseollidin | 69.98 | 0.64 | 1.01 | pass |
| GC | Vestitol | 74.66 | 0.21 | 0.86 | pass |
| GC | 3'-Hydroxy-4'-O-methylglabridin | 43.71 | 0.57 | 1 | pass |
| GC | Glycyrrhetic acid* | 22.05 | 0.74 | 0.1 | failed to pass |
| GC | Echinatin | 66.58 | 0.17 | 0.38 | failed to pass |
| GC | Gancaonin G | 60 | 0.4 | 0.25 | pass |
| GC | Glabranin | 52.9 | 0.31 | 0.97 | pass |
| GC | Glabrene | 46.27 | 0.44 | 0.99 | pass |
| GC | Glabridin | 53.25 | 0.47 | 0.97 | pass |
| GC | Glepidotin A | 44.72 | 0.35 | 0.79 | pass |
| GC | Glycycoumarin | 23.56 | 0.44 | 0.52 | failed to pass |
| GC | Glycyrin | 52.61 | 0.47 | 0.59 | pass |
| GC | Glycyrrhizic acid* | 19.62 | 0.11 | -2.66 | failed to pass |
| GC | Hispaglabridin A | 14.6 | 0.73 | 1.12 | failed to pass |
| GC | Isolicoflavonol | 45.17 | 0.42 | 0.54 | pass |
| GC | Isoliquiritigenin* | 85.32 | 0.15 | 0.44 | failed to pass |
| GC | kaempferol | 41.88 | 0.24 | 0.26 | pass |
| GC | Licochalcone A | 40.79 | 0.29 | 0.82 | pass |
| GC | Licoisoflavone A | 41.61 | 0.42 | 0.37 | pass |
| GC | Licopyranocoumarin | 80.36 | 0.65 | 0.13 | pass |
| GC | Liquiritigenin* | 32.76 | 0.18 | 0.51 | failed to pass |
| GC | Liquiritin* | / | / | / | failed to pass |
| GC | Medicarpin | 49.22 | 0.34 | 1 | pass |
| GC | Glycyrol | 90.78 | 0.67 | 0.71 | pass |
| GC | Pinocembrin | 64.72 | 0.18 | 0.61 | pass |
| GC | Prunetin | 5.41 | 0.24 | 0.65 | failed to pass |
| CH | Stigmasterol* | 43.83 | 0.76 | 1.44 | pass |
| CH | Isorhamnetin | 49.6 | 0.31 | 0.31 | pass |
| CH | Kaempferol | 41.88 | 0.24 | 0.26 | pass |
| CH | α-Spinasterol | 42.98 | 0.76 | 1.28 | pass |
| CH | Petunidin | 30.05 | 0.31 | 0.16 | pass |
| CH | Quercetin | 46.43 | 0.28 | 0.05 | pass |
| CH | Saikosaponin A | 32.39 | 0.09 | -1.95 | failed to pass |
| CH | Isoliquiritigenin* | 85.32 | 0.15 | 0.44 | pass |
| CH | Vanillin | 52 | 0.03 | 0.68 | failed to pass |
| CH | Carvone | 47.43 | 0.03 | 1.34 | failed to pass |
| CH | Saikosaponin D | 34.39 | 0.09 | -1.94 | failed to pass |
| XF | Khell | 33.19 | 0.19 | 1.12 | pass |
| XF | Sugeonyl acetate | 45.08 | 0.2 | 0.72 | pass |
| XF | Isodalbergin | 35.45 | 0.2 | 0.8 | pass |
| XF | Kaempferol | 41.88 | 0.24 | 0.26 | pass |
| XF | Luteolin | 36.16 | 0.25 | 0.19 | pass |
| XF | Chryseriol | 35.85 | 0.27 | 0.39 | pass |
| XF | Quercetin | 46.43 | 0.28 | 0.05 | pass |
| XF | Isorhamnetin | 49.6 | 0.31 | 0.31 | pass |
| XF | Rosenonolactone | 79.84 | 0.37 | 0.72 | pass |
| XF | β-Sitosterol* | 36.91 | 0.75 | 1.32 | pass |
| XF | Stigmasterol* | 43.83 | 0.76 | 1.44 | pass |
| ZQ | Kaempferol | 41.88 | 0.24 | 0.26 | pass |
| ZQ | β-Sitosterol* | 36.91 | 0.75 | 1.32 | pass |
| ZQ | Rutin | 3.2 | 0.68 | -1.93 | failed to pass |
| ZQ | Quercetin | 46.43 | 0.28 | 0.05 | pass |
| ZQ | Syrigin | 14.64 | 0.32 | -1.01 | failed to pass |
| ZQ | Hesperetin | 70.31 | 0.27 | 0.37 | pass |
| ZQ | Hesperidin | 13.33 | 0.67 | -2.03 | failed to pass |
| ZQ | Limonin | 21.3 | 0.57 | -0.15 | failed to pass |
| ZQ | Marmin | 38.23 | 0.31 | 0.14 | pass |
| ZQ | Nobiletin | 61.67 | 0.52 | 1.05 | pass |
| ZQ | Obacunone | 43.29 | 0.77 | 0.01 | pass |
| ZQ | Tangeretin* | 21.38 | 0.43 | 1.23 | failed to pass |
| ZQ | Astragalin | 14.03 | 0.74 | -1.34 | failed to pass |
| ZQ | Luteolin | 36.16 | 0.25 | 0.19 | pass |

DG: *Angelicae Sinensis Radix*; CS: *Paeoniae Radix Rubra*; CX: *Chuanxiong Rhizoma*; TR: *Persicae Semen*; HH: *Carthami Flos*; CH: *Bupleuri Radix*; XF: *Cyperi Rhizoma*; DS: *Salviae Miltiorrhizae Radix et Rhizoma*; YHS: *Corydalis Rhizoma*; JG: *Platycodonis Radix*; ZQ: *Aurantii Fructus*; WY: *Linderae Radix*; NX: *Achyranthis Bidentatae Radix*; SM: *Cimicifugae Rhizoma*; GC: *Glycyrrhizae Radix et Rhizoma*; OB: Oral bioavailability; Caco-2: Caco-cell permeability; DL: Drug-likeness; Screening criteria: OB ≥ 30%, DL ≥ 0.18 and Caco-2 ≥ -0.4. The active compounds which had been verified by UPLC-Q/TOF-MS in QXH were labeled with *.

**Table S2: The active compounds involved in hemorheological abnormality module and coagulopathy module.**

| **Hemorheological abnormality** | | **Coagulopathy** | |
| --- | --- | --- | --- |
| **Herbs** | **Compounds** | **Herbs** | **Compounds** |
| DG | Z-Ligustilide* | DG | Z-Ligustilide* |
| DG | Senkyunolide A | DG | Senkyunolide A |
| DG | Senkyunolide I | DG | Senkyunolide I |
| DG | Z-butylidenephthalide | DG | Z-butylidenephthalide |
| DG | E-butylidenephthalide | DG | E-butylidenephthalide |
| DG | 3-Butylidenephthalide | DG | 3-Butylidenephthalide |
| DG | Coniferyl ferulate | DG | Coniferyl ferulate |
| DG | Stigmasterol* | DG | Stigmasterol* |
| DG | Vanillin | DG | Vanillin |
| DG | β-Sitosterol* | DG | Nicotinic acid |
| DG | Nicotinic acid | DG | Ferulic acid* |
| DG | Ferulic acid* | DG | Caffeic acid* |
| DG | Caffeic acid* | DG | Sedanolide |
| DG | Sedanolide | DG | Folic acid |
| DG | Folic acid | DG | Nodakenin |
| DG | Nodakenin | DG | Chlorogenic acid |
| DG | Chlorogenic acid | DG | Senkyunolide H |
| DG | Senkyunolide H | CS | D-(+)-Catechin |
| CS | D-(+)-Catechin | CS | Ellagic acid |
| CS | Ellagic acid | CS | Lactiflorin* |
| CS | Paeoniflorigenone | CS | Paeoniflorin* |
| CS | Evofolin B | CS | Benzoylpaeoniflorin* |
| CS | Paeonolide | CS | Albiflorin* |
| CS | Paeonol | CS | Paeoniflorigenone |
| CS | Gallic acid | CS | Evofolin B |
| CS | Butylated hydroxytoluene* | CS | Oxypaeoniflorin* |
| CS | Eugenol | CS | Paeonolide |
| CS | Hexanal | CS | Paeonol |
| CX | Ferulic acid* | CS | Gallic acid |
| CX | Caffeic acid* | CS | Butylated hydroxytoluene* |
| CX | Coniferyl ferulate | CS | Eugenol |
| CX | Z-Ligustilide* | CS | Hexanal |
| CX | 3-Butylidenephthalide | CX | Ferulic acid* |
| CX | Senkyunolide A | CX | Caffeic acid* |
| CX | Senkyunolide I | CX | Coniferyl ferulate |
| CX | Senkyunolide H | CX | Z-Ligustilide* |
| CX | Tetramethylpyrazine | CX | 3-Butylidenephthalide |
| CX | Levistilide A* | CX | Senkyunolide A |
| CX | Vanillin | CX | Senkyunolide I |
| CX | Perlolyrine | CX | Senkyunolide H |
| CX | Methyleugenol | CX | Tetramethylpyrazine |
| CX | Sedanolide | CX | Levistilide A* |
| CX | Thymol | CX | Vanillin |
| TR | Amygdalin* | CX | Perlolyrine |
| TR | Prunasin | CX | Methyleugenol |
| TR | Campesterol | CX | Sedanolide |
| TR | 2,3-Didehydrogibberellin A70 | CX | Thymol |
| TR | Gibberellin A119 | TR | Amygdalin* |
| TR | Gibberellin A120 | TR | Prunasin |
| TR | Gibberellin A121-isolactone | TR | 2,3-Didehydrogibberellin A70 |
| TR | Gibberellin A122 | TR | Gibberellin A119 |
| TR | Gibberellin A122-isolactone | TR | Gibberellin A120 |
| TR | Gibberellin A44 | TR | Gibberellin A121-isolactone |
| TR | Gibberellin A7 | TR | Gibberellin A122 |
| HH | Carthamone | TR | Gibberellin A122-isolactone |
| HH | Safflor yellow A | TR | Gibberellin A44 |
| HH | Precarthamin | TR | Gibberellin A7 |
| HH | 6-Hydroxykaempferol | HH | Carthamone |
| HH | Kaempferol | HH | Safflor yellow A |
| HH | Quercetin | HH | Precarthamin |
| HH | Rutin | HH | Safflor yellow B |
| HH | Eriodictyol | HH | 6-Hydroxykaempferol |
| HH | Scutellarin | HH | Kaempferol |
| HH | Scutellarein | HH | Nicotiflorin |
| HH | Acacetin | HH | Quercetin |
| HH | Luteolin | HH | Rutin |
| HH | Rosmarinic acid* | HH | Eriodictyol |
| JG | Luteolin | HH | Scutellarin |
| JG | Cis-dihydroquercetin | HH | Scutellarein |
| JG | Spinasterol | HH | Acacetin |
| JG | Betulin | HH | Luteolin |
| NX | Wogonin | HH | Rosmarinic acid* |
| NX | Oleanolic acid | JG | Luteolin |
| NX | Betaine | JG | Cis-dihydroquercetin |
| NX | Stigmasterol* | JG | Robinin |
| NX | Dibutyl phthalate | JG | Betulin |
| NX | 5-Hydroxymethylfurfural | NX | Wogonin |
| NX | 25R-Inokosterone | NX | Oleanolic acid |
| NX | Ecdysterone | NX | Betaine |
| NX | Chrysophanol | NX | Stigmasterol* |
| WY | Lindestrenolide | NX | Dibutyl phthalate |
| WY | Laurolitsine | NX | 5-Hydroxymethylfurfural |
| WY | Linderane | NX | Ecdysterone |
| WY | Nubigenol | NX | Chrysophanol |
| WY | Boldine | WY | Lindestrenolide |
| WY | Quercetin | WY | Laurolitsine |
| DS | Sugiol | WY | Linderane |
| DS | Dehydrotanshinone II A | WY | Nubigenol |
| DS | Baicalin | WY | Boldine |
| DS | Przewaquinone B | WY | Quercetin |
| DS | Przewaquinone C | DS | Sugiol |
| DS | Danshenol B | DS | Dehydrotanshinone II A |
| DS | Danshenol A | DS | Baicalin |
| DS | Cryptotanshinone* | DS | Przewaquinone B |
| DS | Danshenspiroketallactone | DS | Przewaquinone C |
| DS | Neocryptotanshinone II | DS | Danshenol B |
| DS | Dihydrotanshinone I | DS | Danshenol A |
| DS | Isocryptotanshinone | DS | Cryptotanshinone* |
| DS | Miltirone | DS | Danshenspiroketallactone |
| DS | Neocryptotanshinone | DS | Neocryptotanshinone II |
| DS | Tanshindiol A | DS | Dihydrotanshinone I |
| DS | Tanshindiol B | DS | Isocryptotanshinone |
| DS | Tanshindiol C | DS | Miltirone |
| DS | Tanshinone VI | DS | Neocryptotanshinone |
| DS | Tanshinone IIA* | DS | Tanshindiol A |
| DS | Salvianolic acid B | DS | Tanshindiol B |
| DS | Danshensu | DS | Tanshindiol C |
| DS | Protocatechuic aldehyde | DS | Tanshinone VI |
| DS | Protocatechuic acid | DS | Tanshinone IIA* |
| DS | Caffeic acid* | DS | Salvianolic acid B |
| DS | Rosmarinic acid* | DS | Protocatechuic aldehyde |
| DS | Salvianolic acid A | DS | Protocatechuic acid |
| DS | Tanshinone I* | DS | Caffeic acid* |
| DS | Tanshinone IIB | DS | Rosmarinic acid* |
| YHS | Berberine* | DS | Salvianolic acid A |
| YHS | Coptisine | DS | Tanshinone I* |
| YHS | Cryptopine | DS | Tanshinone IIB |
| YHS | Dihydrochelerythrine | YHS | Berberine* |
| YHS | Dihydrosanguinarine | YHS | Coptisine |
| YHS | Sanguinarine | YHS | Cryptopine |
| YHS | (R)-Canadine | YHS | Dihydrochelerythrine |
| YHS | Tetrahydropalmatine* | YHS | Dihydrosanguinarine |
| YHS | Capaurine | YHS | Sanguinarine |
| YHS | Corydaline | YHS | (R)-Canadine |
| YHS | Corydalmine | YHS | Tetrahydropalmatine* |
| YHS | Corydine | YHS | Capaurine |
| YHS | Dehydrocavidine | YHS | Corydaline |
| YHS | Dehydrocorybulbine | YHS | Corydalmine |
| YHS | Dehydrocorydaline | YHS | Corydine |
| YHS | Dehydrocorydalmine | YHS | Dehydrocavidine |
| YHS | (+)-Isoboldine | YHS | Dehydrocorybulbine |
| YHS | Leonticine | YHS | Dehydrocorydaline |
| YHS | Pseudocoptisine | YHS | Dehydrocorydalmine |
| YHS | Tetrahydrocoptisine | YHS | (+)-Isoboldine |
| YHS | Tetrahydrocorysamine | YHS | Leonticine |
| YHS | Palmatine | YHS | Pseudocoptisine |
| YHS | Protopine | YHS | Tetrahydrocoptisine |
| YHS | Isocorypalmine | YHS | Tetrahydrocorysamine |
| YHS | Bicuculline | YHS | Palmatine |
| YHS | Yuanhunine | YHS | Protopine |
| YHS | Canadine | YHS | Isocorypalmine |
| YHS | Glaucine | YHS | Bicuculline |
| YHS | Oxoglaucine | YHS | Yuanhunine |
| YHS | Columbamine | YHS | Canadine |
| SM | Cimigenoside | YHS | Glaucine |
| SM | Isoferulic acid* | YHS | Oxoglaucine |
| SM | Cimifoetiside III | YHS | Columbamine |
| SM | Dahurinol | SM | Cimigenoside |
| SM | Cimiside E | SM | Isoferulic acid* |
| SM | Cycloartenol | SM | Cimifoetiside III |
| SM | Caffeic acid* | SM | Dahurinol |
| SM | Visamminol | SM | Caffeic acid* |
| SM | Norcimifugin | SM | Cimiside B |
| SM | Cimicifugic acid | SM | Visamminol |
| SM | Visnagin | SM | Norcimifugin |
| SM | Ferulic acid* | SM | Cimicifugic acid |
| SM | 7,8-Didehydrocimigenol | SM | 26-Deoxycimicifugoside |
| SM | Cimigenol | SM | Visnagin |
| GC | 1-Methoxyphaseollidin | SM | Ferulic acid* |
| GC | Vestitol | SM | 7,8-Didehydrocimigenol |
| GC | 3'-Hydroxy-4'-O-Methylglabridin | GC | 1-Methoxyphaseollidin |
| GC | Glycyrrhetic acid* | GC | Vestitol |
| GC | Echinatin | GC | 3'-Hydroxy-4'-O-Methylglabridin |
| GC | Gancaonin G | GC | Glycyrrhetic acid* |
| GC | Glabranin | GC | Echinatin |
| GC | Glabrene | GC | Gancaonin G |
| GC | Glabridin | GC | Glabranin |
| GC | Glepidotin A | GC | Glabrene |
| GC | Glycycoumarin | GC | Glabridin |
| GC | Glycyrin | GC | Glepidotin A |
| GC | Glycyrrhizic acid* | GC | Glycycoumarin |
| GC | Hispaglabridin A | GC | Glycyrin |
| GC | Isolicoflavonol | GC | Glycyrrhizic acid* |
| GC | Isoliquiritigenin* | GC | Hispaglabridin A |
| GC | Licochalcone A | GC | Isolicoflavonol |
| GC | Licoisoflavone A | GC | Isoliquiritigenin* |
| GC | Licopyranocoumarin | GC | Licochalcone A |
| GC | Liquiritigenin* | GC | Licoisoflavone A |
| GC | Liquiritin* | GC | Licopyranocoumarin |
| GC | Medicarpin | GC | Liquiritigenin* |
| GC | Glycyrol | GC | Liquiritin* |
| GC | Pinocembrin | GC | Medicarpin |
| GC | Prunetin | GC | Glycyrol |
| CH | Isorhamnetin | GC | Pinocembrin |
| CH | Kaempferol | GC | Prunetin |
| CH | α-Spinasterol | CH | Isorhamnetin |
| CH | Petunidin | CH | Kaempferol |
| CH | Quercetin | CH | Petunidin |
| CH | Saikosaponin A | CH | Quercetin |
| CH | Saikosaponin D | CH | Saikosaponin A |
| XF | Khell | CH | Saikosaponin D |
| XF | Sugeonyl acetate | XF | Khell |
| XF | Isodalbergin | XF | Sugeonyl acetate |
| XF | Luteolin | XF | Isodalbergin |
| XF | Chryseriol | XF | Luteolin |
| XF | Rosenonolactone | XF | Chryseriol |
| ZQ | Kaempferol | ZQ | Kaempferol |
| ZQ | Rutin | ZQ | Rutin |
| ZQ | Quercetin | ZQ | Quercetin |
| ZQ | Syrigin | ZQ | Syrigin |
| ZQ | Hesperetin | ZQ | Hesperetin |
| ZQ | Hesperidin | ZQ | Hesperidin |
| ZQ | Limonin | ZQ | Marmin |
| ZQ | Marmin | ZQ | Nobiletin |
| ZQ | Nobiletin | ZQ | Obacunone |
| ZQ | Obacunone | ZQ | Tangeretin* |
| ZQ | Tangeretin* | ZQ | Astragalin |
| ZQ | Astragalin | ZQ | Luteolin |
| ZQ | Luteolin |  |  |

DG: *Angelicae Sinensis Radix*; CS: *Paeoniae Radix Rubra*; CX: *Chuanxiong Rhizoma*; TR: *Persicae Semen*; HH: *Carthami Flos*; CH: *Bupleuri Radix*; XF: *Cyperi Rhizoma*; DS: *Salviae Miltiorrhizae Radix et Rhizoma*; YHS: *Corydalis Rhizoma*; JG: *Platycodonis Radix*; ZQ: *Aurantii Fructus*; WY: *Linderae Radix*; NX: *Achyranthis Bidentatae Radix*; SM: *Cimicifugae Rhizoma*; GC: *Glycyrrhizae Radix et Rhizoma*; The active compounds which had been verified by UPLC-Q/TOF-MS in QXH were labeled with *.

**Table S3: The candidate targets involved in hemorheological abnormality module and coagulopathy module.**

| **Hemorheological**  **abnormality** | **Coagulopathy** |
| --- | --- |
| APP | HMOX1 |
| CYP3A4 | NQO1 |
| PTGS1 | MAPK3 |
| PTGS2 | PTGS1 |
| ESR1 | PTGS2 |
| ESR2 | ADRA2C |
| NR1H4 | ADRA2B |
| MMP2 | PRKACA |
| PGR | NOS3 |
| NR3C2 | PRKCG |
| HMGCR | PRKCA |
| APOA1 | PRKCQ |
| NR1H2 | PRKCD |
| NR1H3 | PLAU |
| AR | F10 |
| LDLR | TUBB1 |
| RORA | F3 |
| APOB | KNG1 |
| PPARA | IL6 |
| ABCA1 | TLR4 |
| ALOX15 | F2 |
| APOE | APOE |
| AGT | AGT |
| ADORA1 | F12 |
| IL4 | SYK |
| PIK3CG | PTAFR |
| SERPINE1 | F9 |
| SELP | F7 |
| PECAM1 | PLA2G4A |
| PLAT | PTPN2 |
| CD36 | PIK3CG |
| APOC3 | SERPINE1 |
| ALOX15B | SELP |
| CXCL10 | PECAM1 |
| EDN1 | PLAT |
| JAK2 | CD36 |
| OLR1 | ITGA2B |
| EPO | ALOX12 |
| MAPK14 | ADORA2A |
| SERPIND1 | TBXA2R |
| PPARG | PTPN6 |
| PLA2G1B | EIF6 |
| CHRM5 | CYCS |
| CYP7A1 | EDN1 |
| PRKCH | HGF |
| PTGER4 | JAK2 |
| PRKCE | CD40 |
| F5 | OLR1 |
| AVPR2 | SERPIND1 |
| MTTP | CD40LG |
| CSF1R | PTGER3 |
|  | GATA1 |
|  | F8 |
|  | F2R |
|  | P2RX7 |
|  | PIK3CA |
|  | PIK3CB |
|  | PRKCH |
|  | MPI |
|  | ITGB3 |
|  | PRKCE |
|  | F5 |
|  | HPGDS |
|  | PDGFRA |
|  | HPSE |
|  | FGA |
|  | FGB |
|  | FGG |

**Table S4: The overlapped targets in hemorheological abnormality module and coagulopathy module.**

| **Overlapped targets** |
| --- |
| PTGS1 |
| PTGS2 |
| APOE |
| AGT |
| PIK3CG |
| SERPINE1 |
| SELP |
| PECAM1 |
| PLAT |
| CD36 |
| EDN1 |
| JAK2 |
| OLR1 |
| SERPIND1 |
| PRKCH |
| PRKCE |
| F5 |


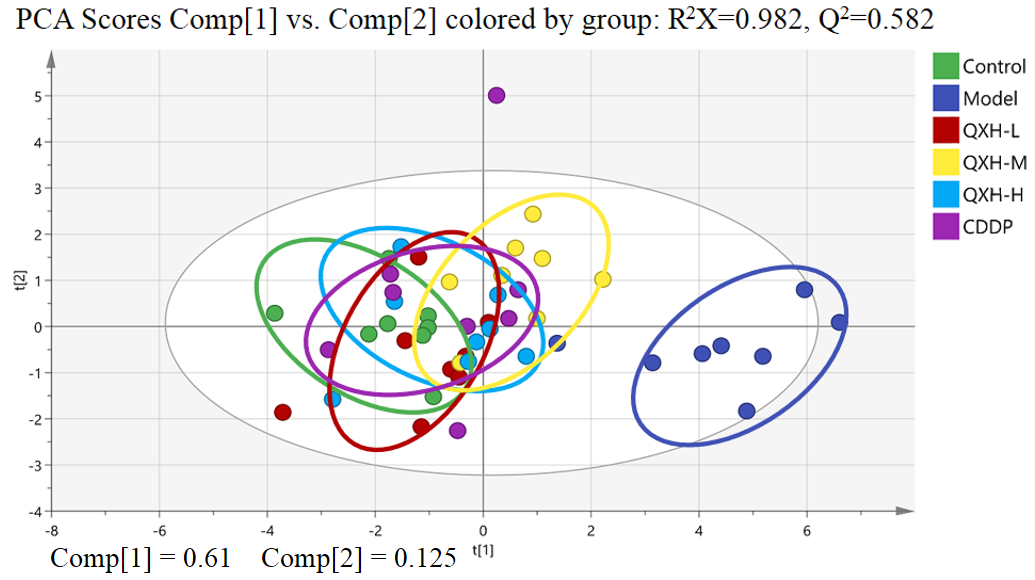


**Figure S1: PCA score plots of hemorheology and coagulation function indexes among all groups.**


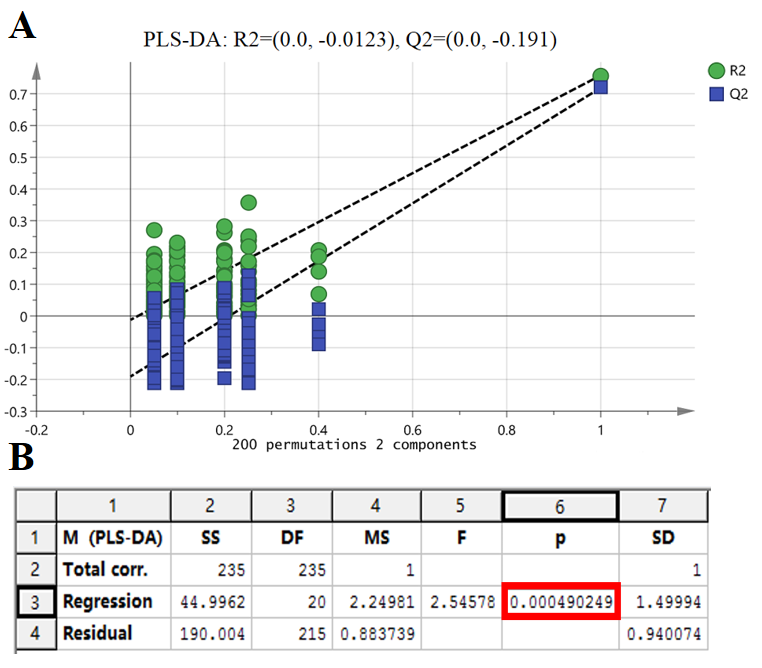


**Figure S2: The permutation tests (n=200) (A) and CV-AVNOA tests (B) for PLS-DA model among all groups.**


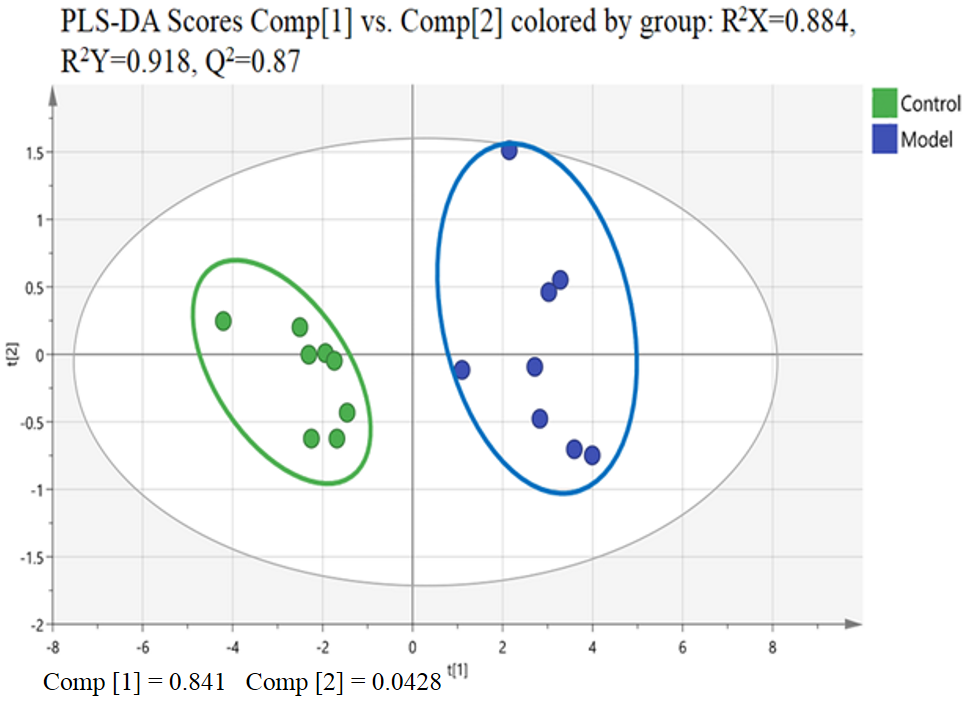


**Figure S3: PLS-DA score plots of hemorheology and coagulation function indexes between control and model groups.**


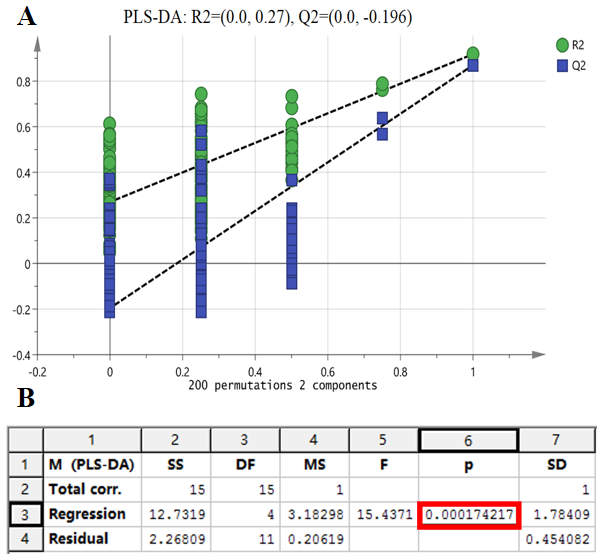


**Figure S4: The permutation tests (n=200) (A) and CV-AVNOA tests (B) for PLS-DA model between control and model groups.**


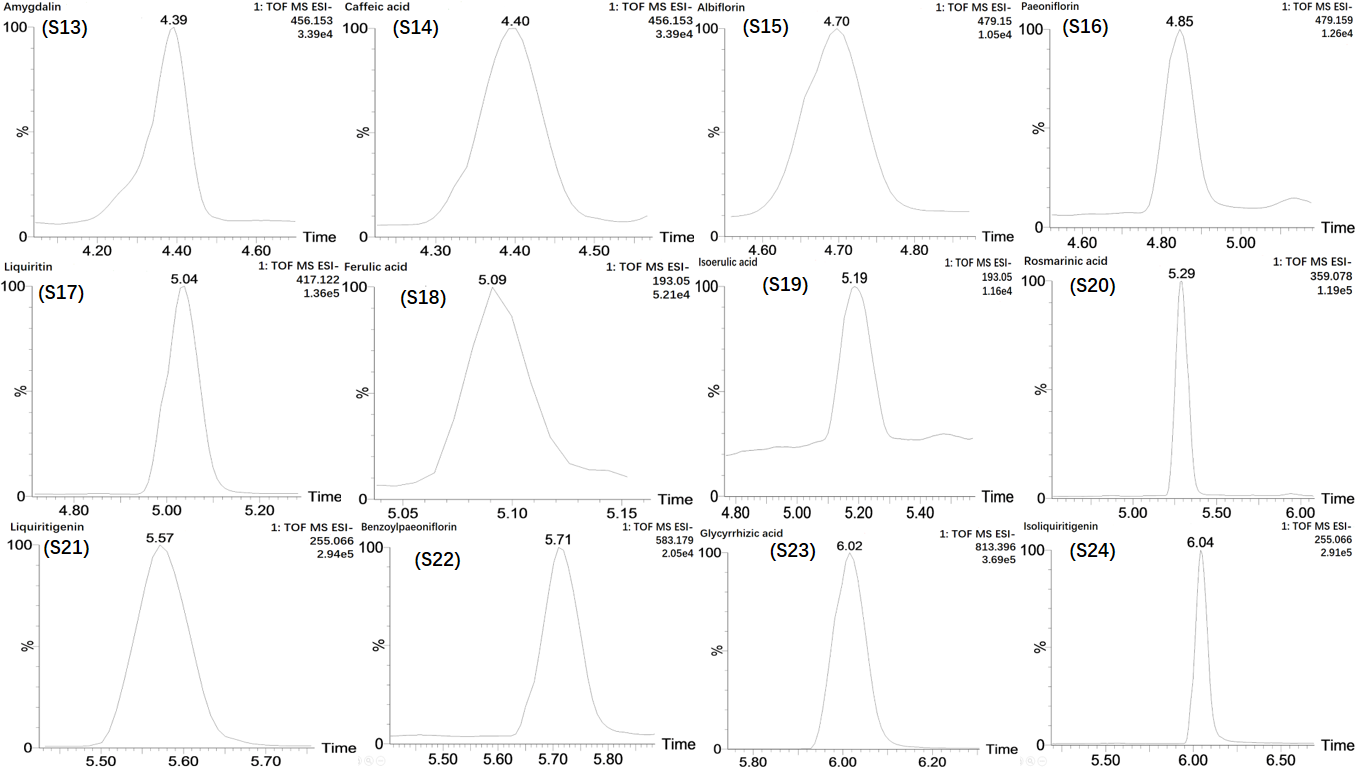

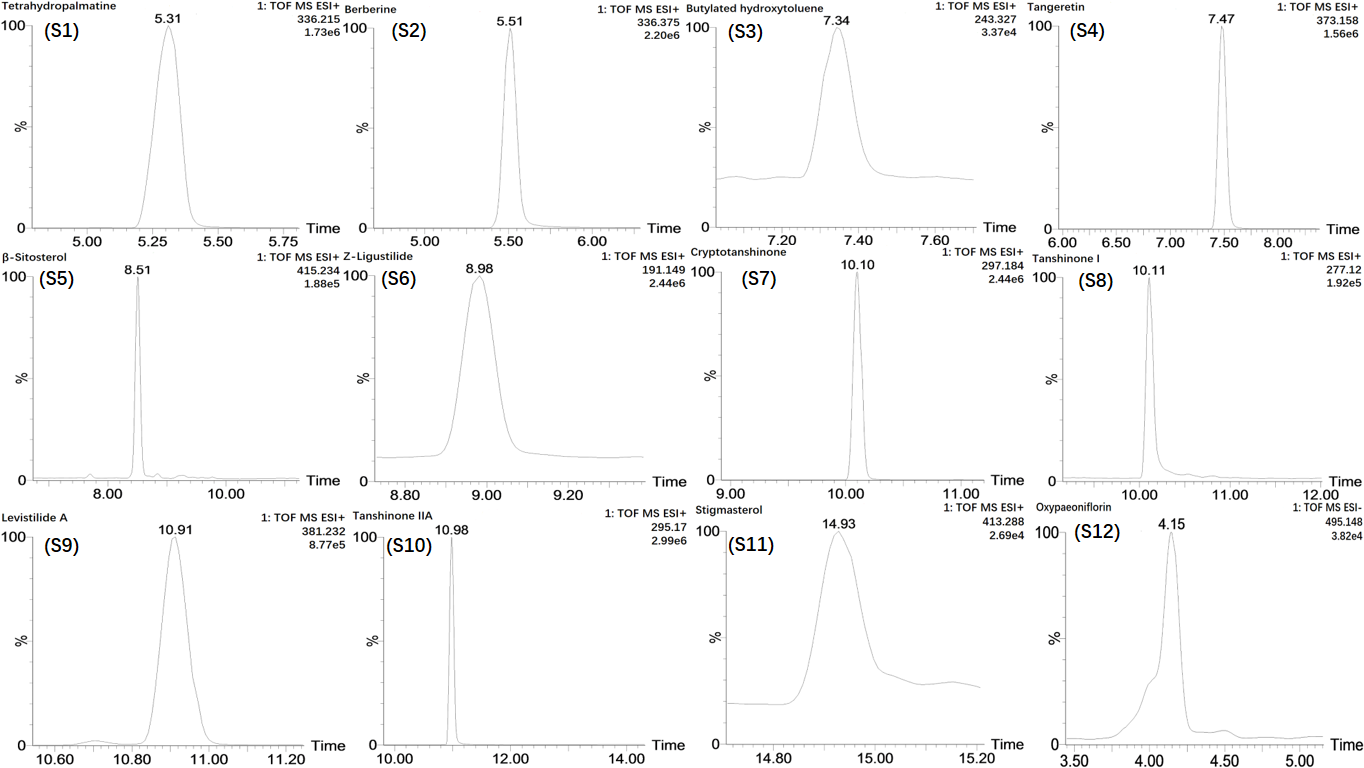


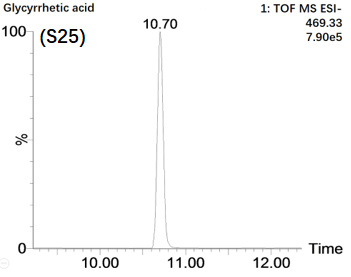


**Figure S5: Base peak intensity chromatograms of 25 standards in positive ion mode or negative ion mode.**


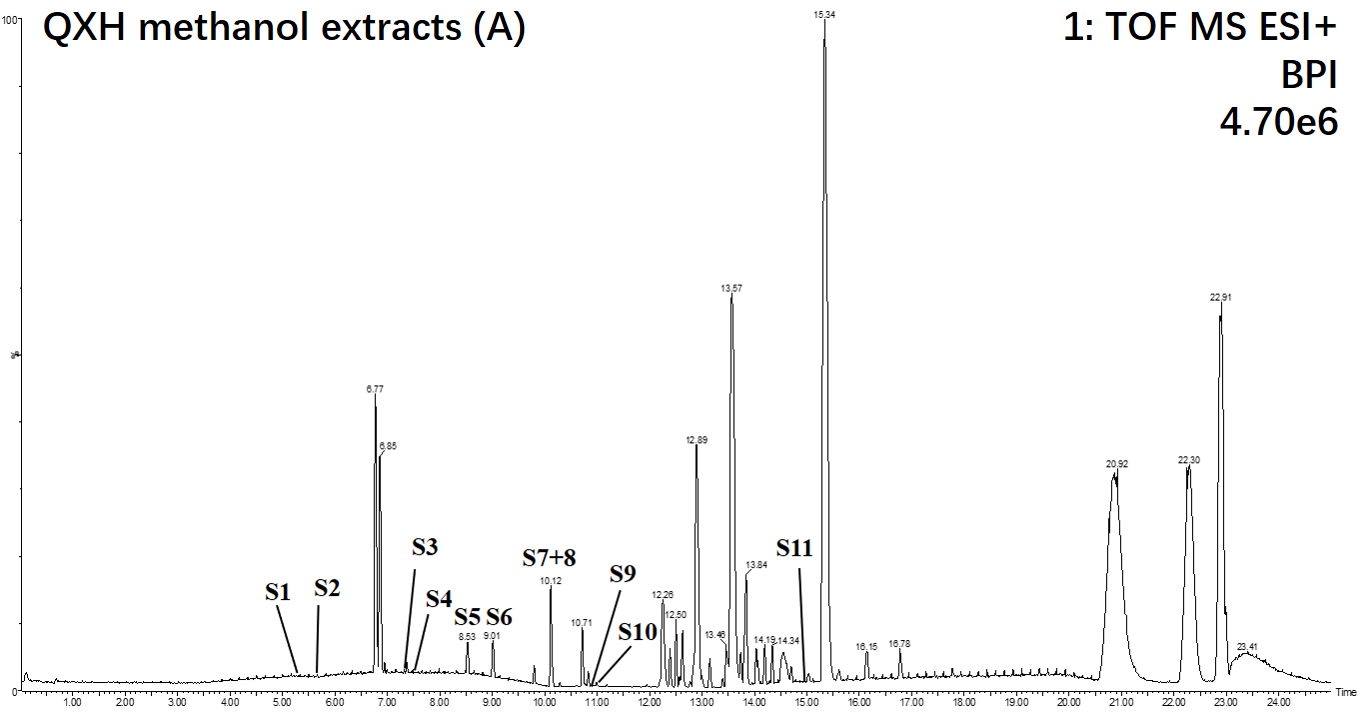

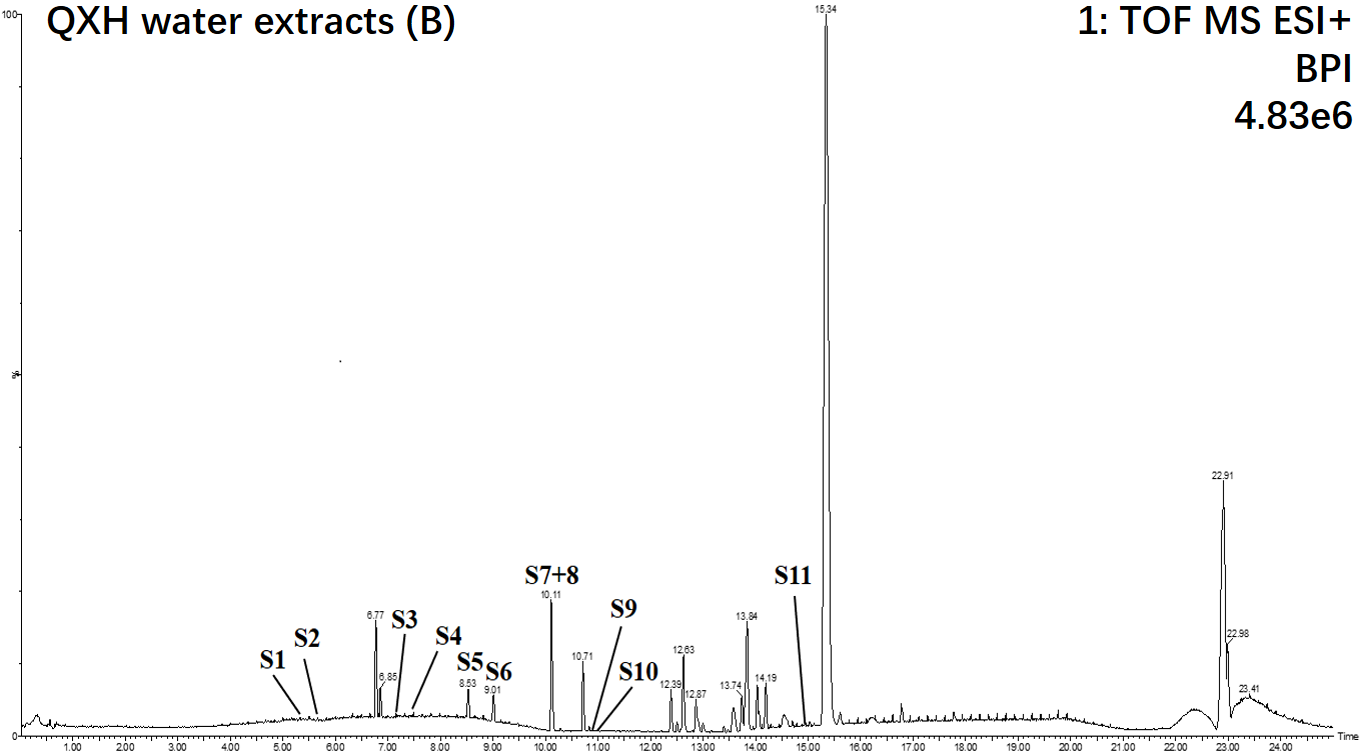


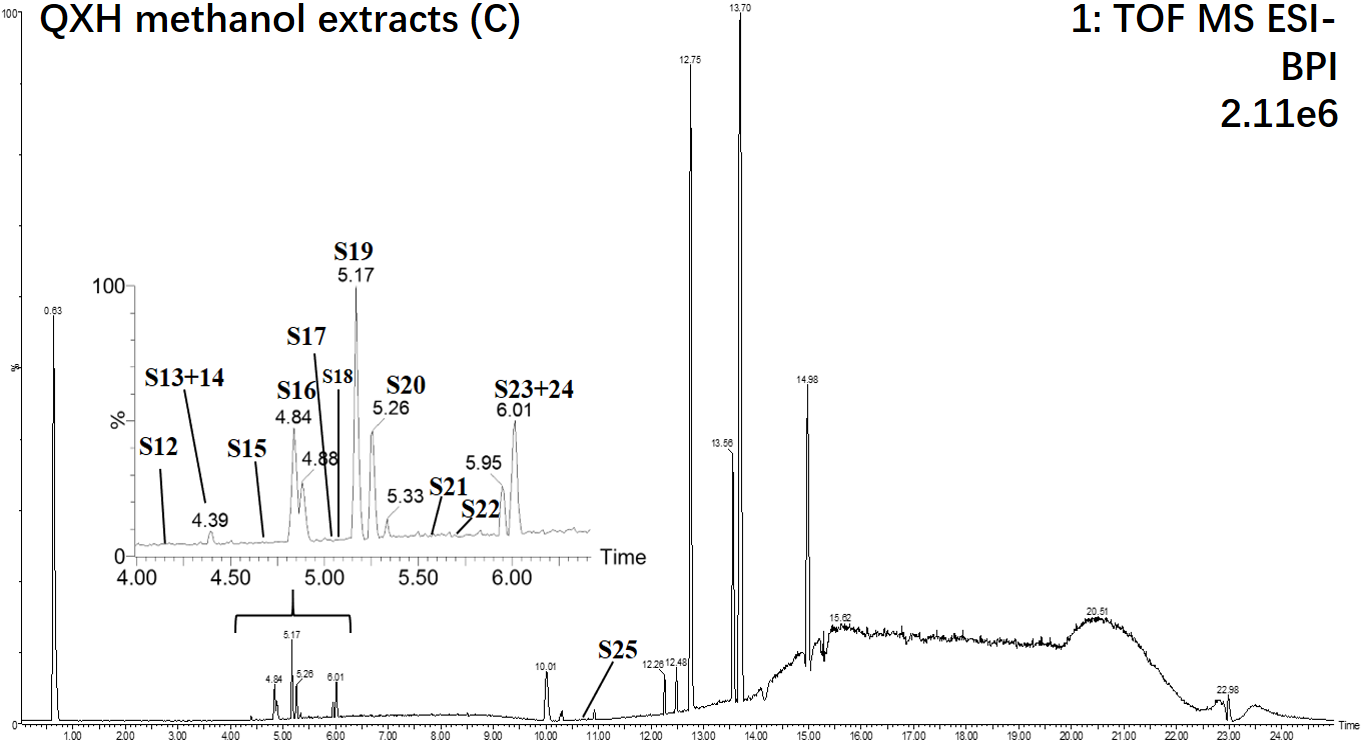


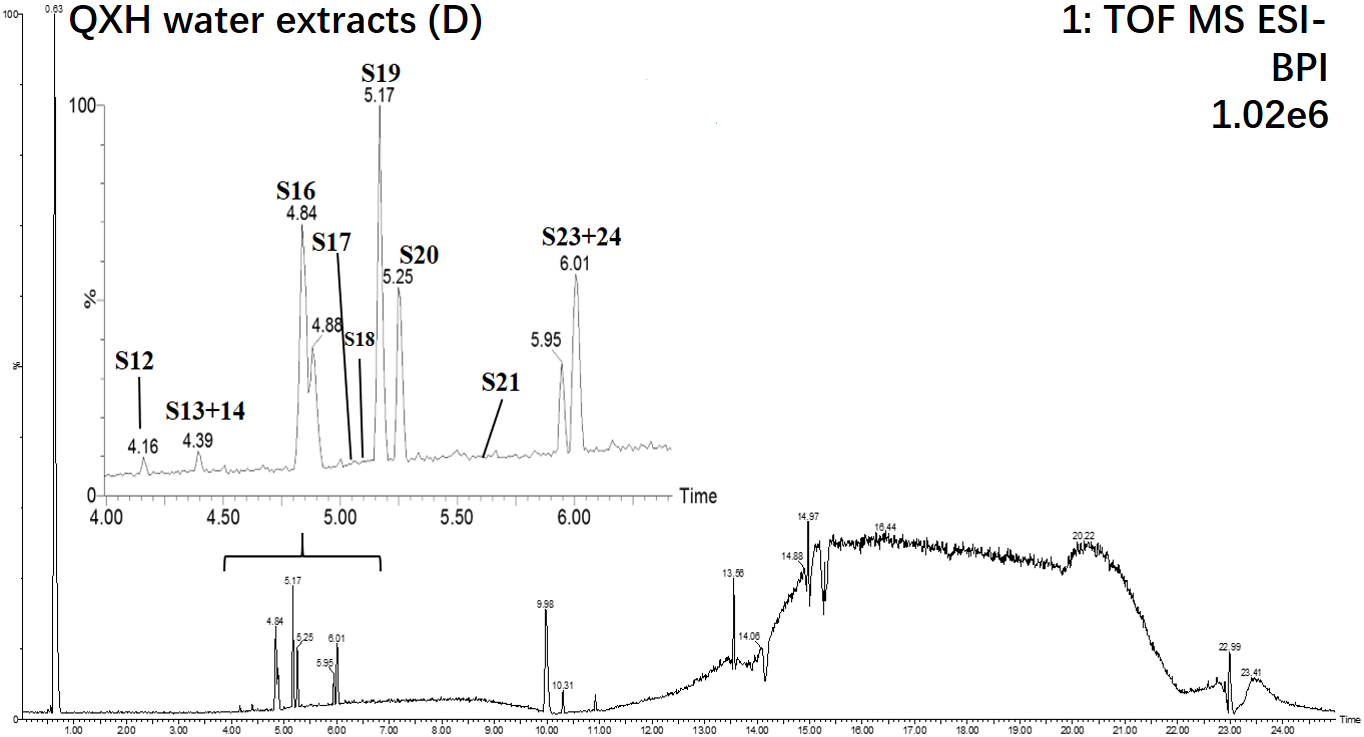


**Figure S6: Base peak intensity chromatograms of QXH methanol extracts (A) in positive ion mode and (C) in negative ion mode, base peak intensity chromatograms of QXH water extracts (B) in positive ion mode and (D) in negative ion mode by UPLC-Q-TOF/MS. S1:** **Tetrahydropalmatine, S2: Berberine, S3: Butylated hydroxytoluene, S4: Tangeretin, S5: β-Sitosterol, S6: Z-Ligustilide, S7:** **Cryptotanshinone, S8: Tanshinone I, S9: Levistilide A, S10: Tanshinone IIA, S11: Stigmasterol, S12: Oxypaeoniflorin, S13: Amygdalin, S14: Caffeic acid, S15: Albiflorin, S16: Paeoniflorin, S17: Liquiritin, S18: Ferulic acid, S19: Isoferulic acid, S20: Rosmarinic acid, S21: Liquiritigenin, S22: Benzoylpaeoniflorin, S23: Glycyrrhizic acid, S24:** **Isoliquiritigenin, S25: Glycyrrhetic acid.**

**
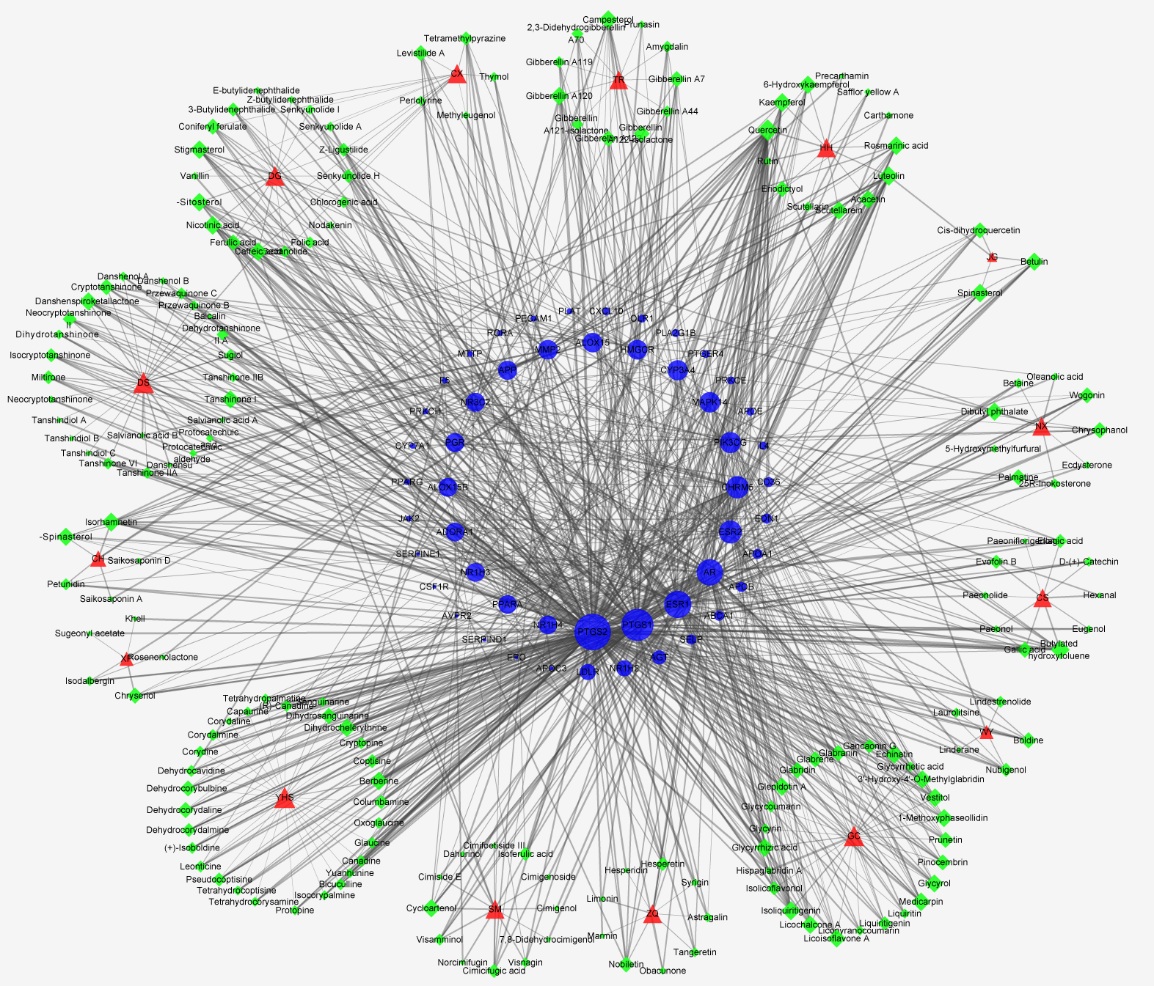
Figure S7: The H_H_-C_H_-T_H_ network of QXH (hemorheological abnormality module; red node: herbs, green node: compounds and blue node: targets) (the original picture for Figure 3).**


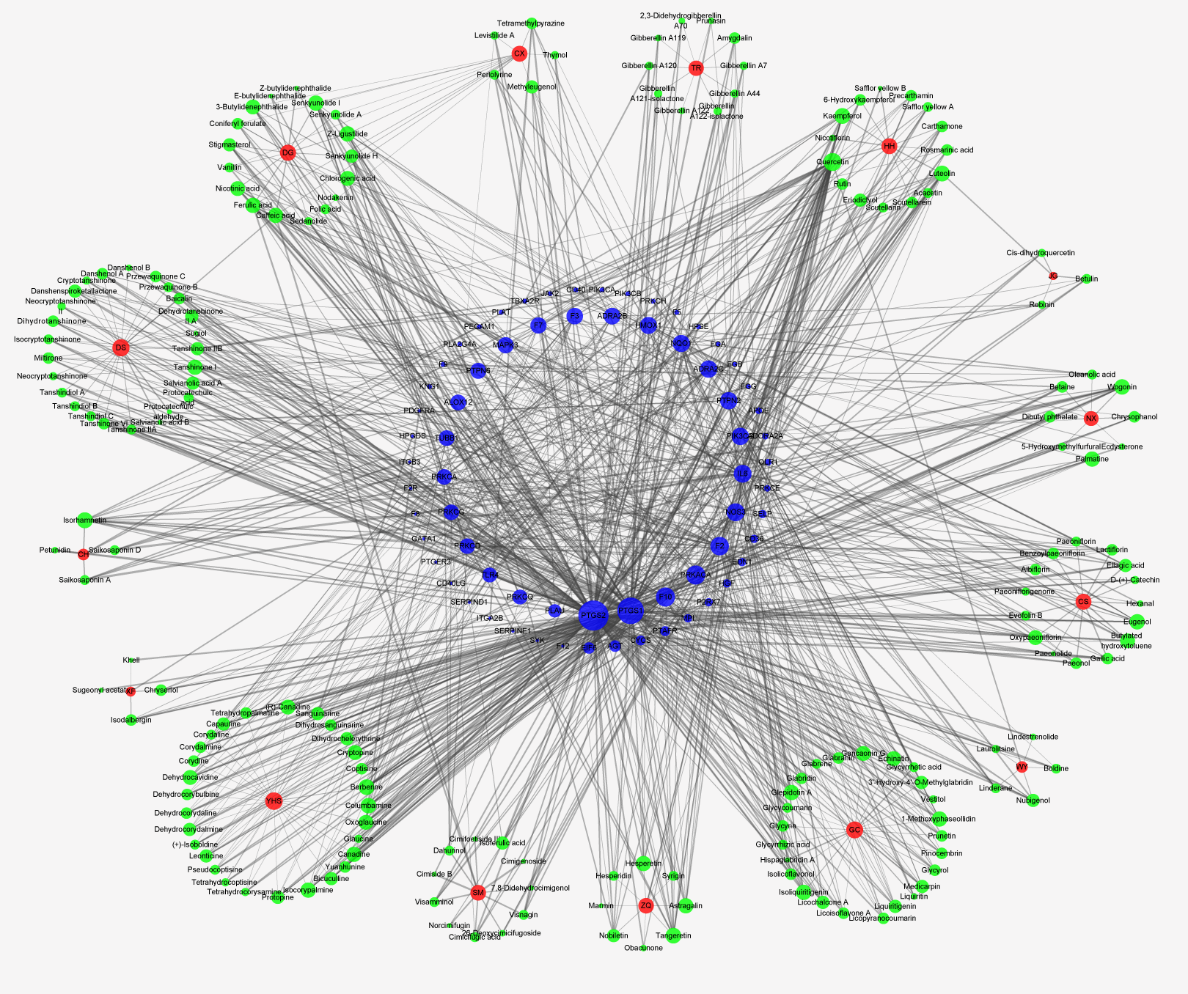


**Figure S8: The H_C_-C_C_-T_C_ network of QXH (coagulopathy module, red node: herbs, green node: compounds and blue node: targets) (the original picture for Figure 5).**
